# A Protocol for Lab‐Scale Production of ^13^C‐Labeled Yeast Extract as Internal Standard for Metabolomics and Quantification of Intracellular Metabolites

**DOI:** 10.1002/cpz1.70470

**Published:** 2026-11-03

**Authors:** Miriam Cammaert, Rosalie Wouters, Jitske van Ede, Erik de Hulster, Christiaan Mooiman, Patricia van Dam, Martin Pabst, Walter van Gulik, Pascale Daran‐Lapujade

**Affiliations:** ^1^ Department of Biotechnology Delft University of Technology Delft the Netherlands; ^2^ These authors contributed equally to this work

**Keywords:** ^13^C‐labeled yeast extract, Fed‐batch fermentation, Internal standards, Mass spectrometry, Metabolomics

## Abstract

Metabolomics enables the profiling of small‐molecule metabolites, thereby capturing the biochemical state of a living organism at a given moment and making it possible to monitor the organism's cellular responses to stimuli. This technique has become a powerful tool in pharmaceutical research, the food industry, and microbial research. Metabolomics aims to obtain an unbiased metabolic profile; however, this is complicated by compound instability, the requirement for complex and often extensive sample processing, and nonlinear responses in mass spectrometry. Therefore, correcting for metabolite loss and mass‐spectrometry‐related artifacts is essential; this is typically achieved through relative quantification against an isotopically labeled internal standard for each metabolite of interest. This article describes a method to produce ^13^C‐labeled yeast extract and its use as internal standard for metabolomics. More specifically, it provides step‐by‐step protocols for the fed‐batch fermentation, quenching, metabolite extraction, and LC‐MS and GC‐MS characterization of the internal standard. It also includes a protocol explaining how to use the internal standard for the quantification of metabolites in yeast samples. © 2026 The Author(s). *Current Protocols* published by Wiley Periodicals LLC.

**Basic Protocol 1**: Trial run—fed‐batch fermentation

**Support Protocol 1**: Dry weight determination

**Basic Protocol 2**: Quenching and washing of the cell samples and metabolite extraction

**Basic Protocol 3**: Identification of intracellular metabolites using LC‐MS

**Basic Protocol 4**: Identification of intracellular metabolites using GC‐MS

**Basic Protocol 5**: Real run—fed‐batch fermentation

**Basic Protocol 6**: LC‐MS characterization of the internal standard

**Basic Protocol 7**: GC‐MS characterization of the internal standard

**Basic Protocol 8**: Quantification of metabolites in yeast samples

## INTRODUCTION

Metabolic profiling is a powerful tool for characterizing biological systems. By detecting and quantifying many intracellular metabolites simultaneously in one biological sample, metabolomics captures the biochemical state of a living organism at a given moment and makes it possible to monitor its cellular responses to stimuli. Metabolomics is an established technique that is used in both fundamental and applied research in various fields, including pharmaceutical research (Wishart, 2016), the food industry (Li et al., 2021), and microbial research (Sailwal et al., 2020; Tang, 2011).

The aim of metabolomics is to obtain an unbiased *in vivo* snapshot of the metabolic profile of a living organism. This aim is, however, difficult to achieve because metabolites can be unstable and be lost during the extensive processing required to quench metabolic reactions and extract metabolites from cells and tissues (Canelas et al., 2009). Furthermore, differences in sample matrices between organisms can cause variation in ionization efficiency and signal suppression or enhancement in mass spectrometry (MS; Panuwet et al., 2016). Finally, MS‐based quantification of metabolite levels is error prone because high concentrations of salts or organic compounds in the sample can lead to ion suppression in the electrospray ionization step, causing a nonlinear response of the metabolite levels (Gowda & Djukovic, 2014). These technical challenges can be mitigated by using a method known as isotope dilution MS (ID‐MS; Mashego et al., 2004; Wu et al., 2005). The principle of ID‐MS is based on the addition of a stable isotopically labeled internal standard for every metabolite of interest. ID‐MS thereby allows quantification relative to the internal standard rather than absolute quantification, correcting for metabolite loss and the effects of ion suppression.

Internal standards—typically metabolites isotopically labeled with ^13^C or ^15^N—must meet several criteria. First, the internal standard should be uniformly isotopically labeled. Second, the internal standard should mimic the metabolite profile of the sample to maximize the number of quantifiable metabolites. Third, the internal standard should be readily available and cost effective. Finally, the internal standard should contain metabolites at concentrations closely matching those in the target sample to ensure accurate quantification.

These internal standards can be commercially available or “homemade.” Although commercially available internal standards offer the benefit of quality control, they are at risk of discontinuation by their manufacturers. Furthermore, commercial internal standards are generic and not optimized for the organism and/or condition of interest, which may hinder the accurate measurement of crucial metabolites. These caveats can be circumvented by producing in‐house isotopically labeled internal standard.

The in‐house production of internal standards requires several decisions aimed at optimizing the quality of the standard in terms of metabolite diversity, abundance, and preservation during processing. These choices include the organism (Seike et al., 2025) and the type of (labeled) substrate, depending on the metabolic profile of interest. Furthermore, the type of culture (e.g., batch, fed‐batch, or chemostat) should minimize ^12^C contamination and maximize biomass yield on substrate, defined as grams of dry cell weight produced per gram of substrate consumed. The harvesting and extraction procedure should be chosen as to minimize the degradation and leakage of metabolites. Finally, the choice of analytical methods should take into consideration the metabolites’ chemical properties and their concentration range (Fig. 1).

**Figure 1:**
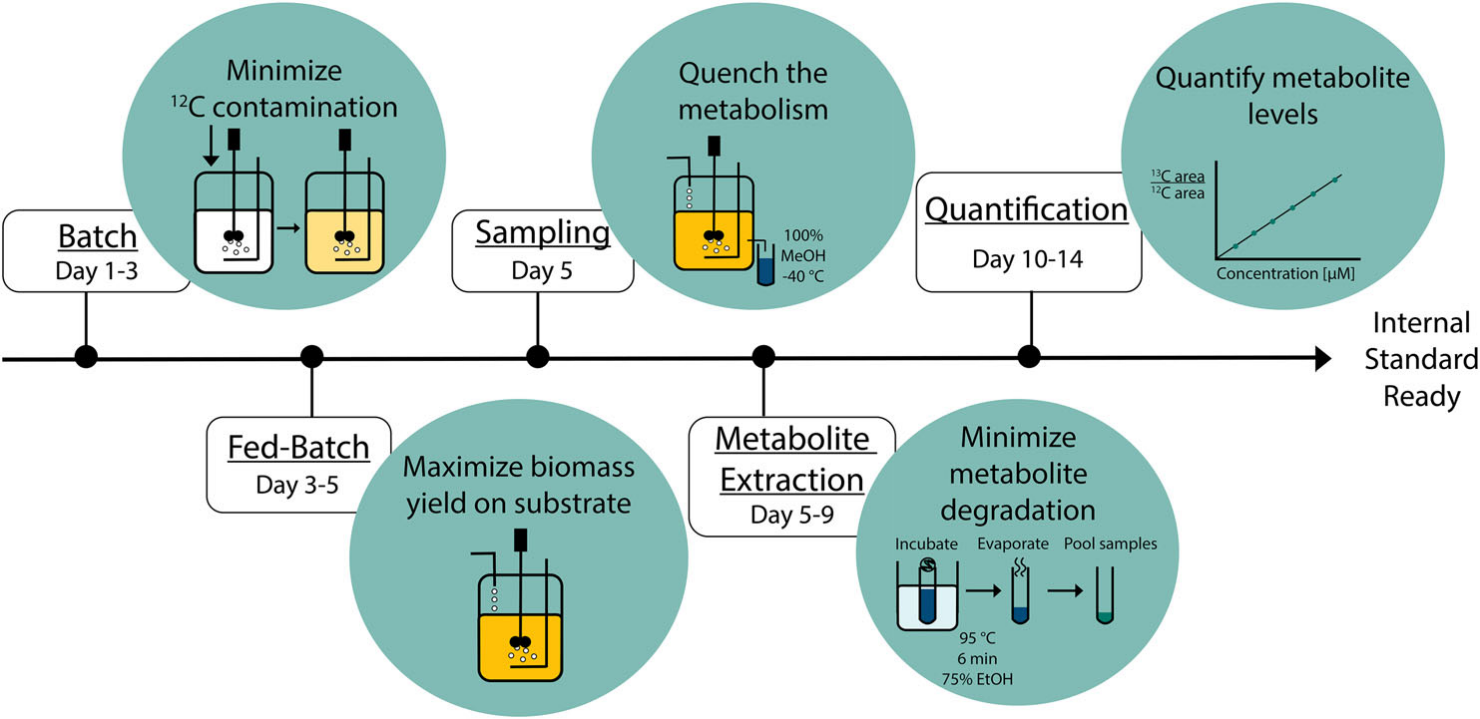
Timeline for in‐house production of ^13^C‐labeled internal standard.

The present study describes a robust procedure for the in‐house production of internal standards, based on fully ^13^C‐labeled yeast biomass. The protocols outline the cultivation of fully labeled *Saccharomyces cerevisiae* in a high‐density fed‐batch process, rapid harvesting and quenching of the biomass, methanol‐based metabolite extraction, and quantification of metabolites in the resulting internal standard using liquid chromatography and gas chromatography coupled to mass spectrometry (LC‐MS and GC‐MS, respectively). The final protocol describes how to use internal standards to quantify intracellular intermediates in NAD^+^ biosynthesis. Although this method focuses on *S*. *cerevisiae*, the internal standard described is relevant for all types of organisms, particularly for the most commonly investigated metabolic pathways (e.g., within the Embden‐Meyerhof‐Parnas [EMP] pathway of glycolysis, the tricarboxylic acid [TCA] cycle, the pentose phosphate pathway, etc.). Furthermore, metabolites absent in *S. cerevisiae* can be added in labeled form to the internal standard if desired. The presented workflow highlights key aspects for the production of internal standards that can be customized to suit different experimental conditions, lab capacities, and microbes.

## STRATEGIC PLANNING

Note that these protocols require basic knowledge of microbial physiology and fermentation technology (Heijnen, 2009) and access to an analytical chemistry facility.

The production process of ^13^C‐labeled internal standards must meet two main requirements: ensure full isotopic labeling of the microbial biomass and maximize the biomass yield on substrate to minimize production costs.

To obtain fully labeled biomass, all non‐labeled sources that might contaminate the metabolites must be removed. In *S. cerevisiae*, isotopic dilution may occur through the incorporation of unlabeled carbon atoms originating from vitamins into intracellular metabolites. To prevent isotopic dilution, pantothenic acid, nicotinic acid, myoinositol, pyridoxine, and 4‐aminobenzoic acid are omitted from the growth medium. These compounds serve as precursors for important cellular metabolites and biomass components: for example, nicotinic acid contributes to the pyridine ring of NAD(P) (Croft et al., 2020), pantothenic acid is a precursor of coenzyme A (Webb et al., 2004), and myo‐inositol is incorporated into membrane phospholipids (Henry et al., 2012). The presence of these vitamins would thus result in mixed isotopologue distributions and reduced ^13^C enrichment of intracellular metabolites. In contrast, biotin and thiamine are included in the medium because they function as cofactors without contributing carbon atoms to the metabolites of interest.

The omission of these medium components reduces the growth rate, which must nevertheless remain sufficient to support controlled and physiologically relevant cultivation conditions. The selected growth rate should allow accurate substrate feeding within the operational range of the feeding system, thereby preventing feast‐famine dynamics, and should avoid impractical long fermentation times considering the availability of laboratory equipment. In addition, the physiological state of the cells should remain relevant for the intended use of the resulting ^13^C‐labeled yeast extract. In the present protocol, *S. cerevisiae* was cultivated at a specific growth rate of 0.05 hr^−1^, corresponding to 12.5% of the maximum growth rate in the presence of all vitamin supplements of 0.4 hr^−1^.

For vitamins that cannot be omitted but might end up contributing carbon, we advise adding commercial ^13^C‐labeled versions to the medium. The same principle applies to other potentially essential nutrients, such as amino acids. Another measure to limit both contamination by unlabeled carbon and the loss of substrate is to inoculate the batch fermentation with a very small amount of unlabeled biomass. Typically, bioreactors are inoculated at 1‐5% (v/v) of the working volume using a preculture of the same cultivation condition. However, preparing precultures with labeled glucose would result in the loss of a significant amount of costly [^13^C]glucose. The bioreactors can be inoculated with unlabeled biomass provided that it represents a minute fraction of the final total biomass. This is achieved by inoculating the batch cultures with a single 1‐ml cryovial of unlabeled *S. cerevisiae*.

To maximize the biomass yield on substrate, it is preferable to favor the most efficient metabolic mode in terms of ATP yield on substrate, which is typically respiration. Consequently, fermentative substrate dissimilation should be prevented or minimized. For instance, *S. cerevisiae* is a Crabtree‐positive yeast that ferments glucose when supplied in excess, even in the presence of excess oxygen, leading to decreased biomass yield on carbon substrate (*Y*_x/s_) as compared to fully respiratory growth (*Y*_x/s_ respiration of 0.5 g_biomass_/g_glucose_ versus *Y*_x/s_ fermentation of 0.1 g_biomass_/g_glucose_; de Deken, 1966; van Dijken et al., 2000). To ensure fully respiratory growth, aerobic glucose limitation is required, which can be reached using glucose‐limited fed‐batch cultures. Conversely, Crabtree‐negative yeasts do not require glucose‐limited conditions, meaning that batch fermentations with excess carbon source can be used, provided that there is sufficient oxygen to prevent oxygen limitation.

It is also important to choose growth conditions, medium, and gas supply for the production of the internal standards that mimic as closely as possible the conditions under which the experiments will be performed. For instance, internal standards produced with glucose as carbon source will not contain labeled intermediates of the Leloir pathway that are produced only when galactose is used as carbon source.

Upon harvesting, extracellular glucose and oxygen—key regulators of metabolism—are taken up by the cells and depleted within milliseconds in the samples (Koning & Dam, 1992), leading to rapid alterations in intracellular metabolite levels, particularly for metabolites with turnover times in the order of seconds. As a result, any delay between harvesting and quenching may compromise the metabolic profile. Therefore, a fermentor equipped with a rapid bottom‐harvesting port is necessary to enable rapid harvesting (Lange et al., 2001). Given the sensitivity of glucose‐limited cultures to glucose and oxygen perturbations, rapid harvesting combined with instantaneous quenching is essential to ensure reliable metabolic profiling. Quenching is an organism‐dependent method that requires careful optimization to prevent leakage of metabolites in the extracellular matrix and their degradation. Instantaneous quenching of various yeast species, including *S. cerevisiae*, *Kluyveromyces marxianus*, and *Pichia pastoris* (recently renamed *Komagataella phaffii*; Bolten & Wittmann, 2008), can be achieved using −40°C methanol (Canelas et al., 2008).

After the cells have been quenched in ice‐cold methanol, the samples are further processed, first to separate intracellular from extracellular metabolites. This is followed by metabolite extraction using boiling ethanol (Canelas et al., 2009), after which the ethanol is removed by vacuum evaporation. The resulting residue cell extract is resuspended in water, filtered, and subsequently processed either by solid‐phase extraction (SPE) or by derivatization in preparation for metabolomic analysis using LC‐MS or GC‐MS, respectively.

The basic protocols are separated into those for a trial run and a real run. Performing a trial run is strongly recommended to evaluate microbial growth, biomass concentration, and physiology under the modified culture conditions. The trial run should be conducted using [^12^C]glucose, which significantly reduces costs and permits sampling in time to assess the fermentation profile. The final biomass concentration achieved during the batch phase is critical data to define the optimal glucose feeding rate for the fed‐batch phase. Overfeeding will result in respiro‐fermentative metabolism and loss of [^12^C]glucose in the form of ethanol, glycerol, and organic acids, whereas underfeeding leads to slow growth rate and associated lower biomass yield on substrate due to maintenance requirements (see Support Protocol 1; Verduyn, 1991). Additionally, the trial run provides the perfect opportunity to practice the rapid harvesting protocol, as well as the subsequent sample processing and metabolite extraction. Given the sensitivity of metabolic profiles to environmental changes, teamwork and a streamlined workflow are essential. After the fermentation and sample processing, at least one unlabeled metabolite sample should be analyzed to confirm that the metabolite profile of interest is obtained under the tested conditions. These validation steps form the foundation for a successful ^13^C‐labeling experiment, during which intermediate sampling is typically avoided because of the high cost of isotopically labeled substrates.

This article describes first how to perform the trial run for the fed‐batch (Basic Protocol 1), followed by sample processing and metabolite extraction (Basic Protocol 2) and verification of the metabolite profile of this trial run (Basic Protocols 3 and 4). The article then describes how to perform the fed‐batch process with [^13^C]glucose (Basic Protocol 5) and how to characterize the internal standard (Basic Protocols 6 and 7). The last protocol (Basic Protocol 8) describes how to quantify metabolites in yeast samples from shake flask cultures using the internal standard.

*NOTE*: To allow the samples to be handled as quickly as possible for harvesting and sample processing (Basic Protocols 1 and 2), gathering a team of at least six people for the harvesting day is convenient as a means to distribute the tasks. Two persons are then responsible for the harvesting, one person adjusts the feeding profile, two persons are responsible for the sample processing, and one person is responsible for changing the centrifugation rotors.

## Basic Protocol 1 TRIAL RUN—FED‐BATCH

It is strongly recommended that one or more trial run(s) with unlabeled glucose be performed. During the trial run, you will prepare and execute an aerobic batch and fed‐batch fermentation, validate the fed‐batch feeding profile, test the rapid harvesting setup, and produce samples for optimization of the analytical pipeline. This protocol describes how to perform an aerobic batch and fed‐batch fermentation in a bioreactor. The growth performance of *S. cerevisiae* is monitored via sampling for biomass and extracellular metabolites and via continuous gas analysis. These data will indicate whether the culture conditions are optimal for a high biomass yield on substrate. Finally, biomass will be harvested from the bioreactors in large quantities using a rapid harvesting device, providing the opportunity to further optimize the rapid harvesting routine if needed. Once this protocol has been successfully performed, you are ready to continue to Basic Protocol 2 (quenching and washing of the cell samples and metabolite extraction).

### Materials

Medium and chemicals:
  Fed‐batch medium (see recipe), preferably prepared fresh on the day of use
  Batch salt base (see recipe)
  pH titration solution (base): 4 M NH_4_OH (see recipe)
  pH calibration solution, pH 4 and 7
  [^12^C]Glucose solution (see recipe)
*Saccharomyces cerevisiae* strain CEN.PK113‐7D or other strain of interest, in 30% (v/v) glycerol (1 ml)
  Demineralized (demi) water, sterile
  Vitamin solution (see recipe)
  Antifoam C solution (see recipe)
  Methanol (HPLC grade, >99.9%), prechilled to −40°C
Bioreactor equipment:
  Fully equipped fermentor with a working volume up to 1.25 liters, including bottom rapid‐harvesting port and several pumps, connected to ez‐Control (e.g., Applikon, Getinge, Delft, the Netherlands)
  Norprene (Masterflex) tubing
  Pump, 0.02‐0.5 ml/min range (for fed‐batch feed)
  pH probe, sterilizable and calibrated between pH 4 and 7
  Dissolved oxygen (DO) probe, sterilizable
  Platinum temperature sensor
  Fixed‐speed pump (20 rpm), with Norprene 13 or 14 tubing, allowing a flow of 1.2‐5.2 ml/min (for antifoam and base)
  Pressure controller capable of reaching 0.3 bar overpressure on the bioreactor
  NGA 2000 Rosemount gas analyzer (e.g., Emerson, St. Louis, MO)
  Scale connected to the control system to log the decrease of fed‐batch medium in the bottle (up to 6200 g)
  Glass shake flask with outlet tube at the bottom
Harvesting equipment:
  1‐liter Schott bottles
  At least three screw caps for Schott bottles with two inlets/outlets
  Scale capable of measuring up to 6200 g (to measure the volume of harvested culture)
  Freezer, set at −40°C
  1‐liter Schott bottles each containing 450 ml methanol and a stir bar, and with a volume indicator at either 550 ml or 575 ml (depending on the harvesting schedule), stored in −40°C freezer
  Magnetic stir plate
  Stir bar
  Tabletop centrifuge, maximum speed 17,000 × *g*
General lab equipment:
  Cryo and nitrile gloves
  HPLC vials
  Agilent 1260 HPLC, equipped with a Bio‐Rad HPX 87H column

Additional reagents and equipment for dry weight determination (see Support Protocol 1)

*CAUTION*: NH_4_OH has a high vapor pressure, and the gas is toxic. Always work in a fume hood, and do not sterilize this solution; rather, autoclave a bottle with water and add NH_4_OH in a fume hood afterwards.

*CAUTION*: When working with high‐molarity acids and bases, please take the required precautions, including wearing protective clothing, glasses, and gloves.

*CAUTION*: Methanol is a neurotoxic compound and can penetrate the skin. Always wear (nitrile) gloves when handling methanol and work as much as possible in the fume hood. Try to minimize the steps performed outside the fume hood.

*CAUTION*: The fermentor is operated under pressure at 0.3 bar. Do not open any ports and/or valves without first depressurizing the reactor. Sudden release of pressure may cause injury or equipment damage.

*CAUTION*: Ensure that the gas analyzer used for off‐gas CO_2_ monitoring can detect isotopically labeled CO_2_. Infrared (IR)‐based sensors may not accurately measure ^13^CO_2_ because its absorption spectrum is shifted relative to ^12^CO_2_ because of the higher isotopic mass. Using an analyzer calibrated only for ^12^CO_2_ can therefore lead to significant underestimation or misinterpretation of CO_2_ levels.

#### Bioreactor preparation

1 (Deep) clean a 2‐liter rapid‐harvesting fermentor and replace all norprene (Masterflex) tubing with fresh tubing (Fig. 2).

**Figure 2:**
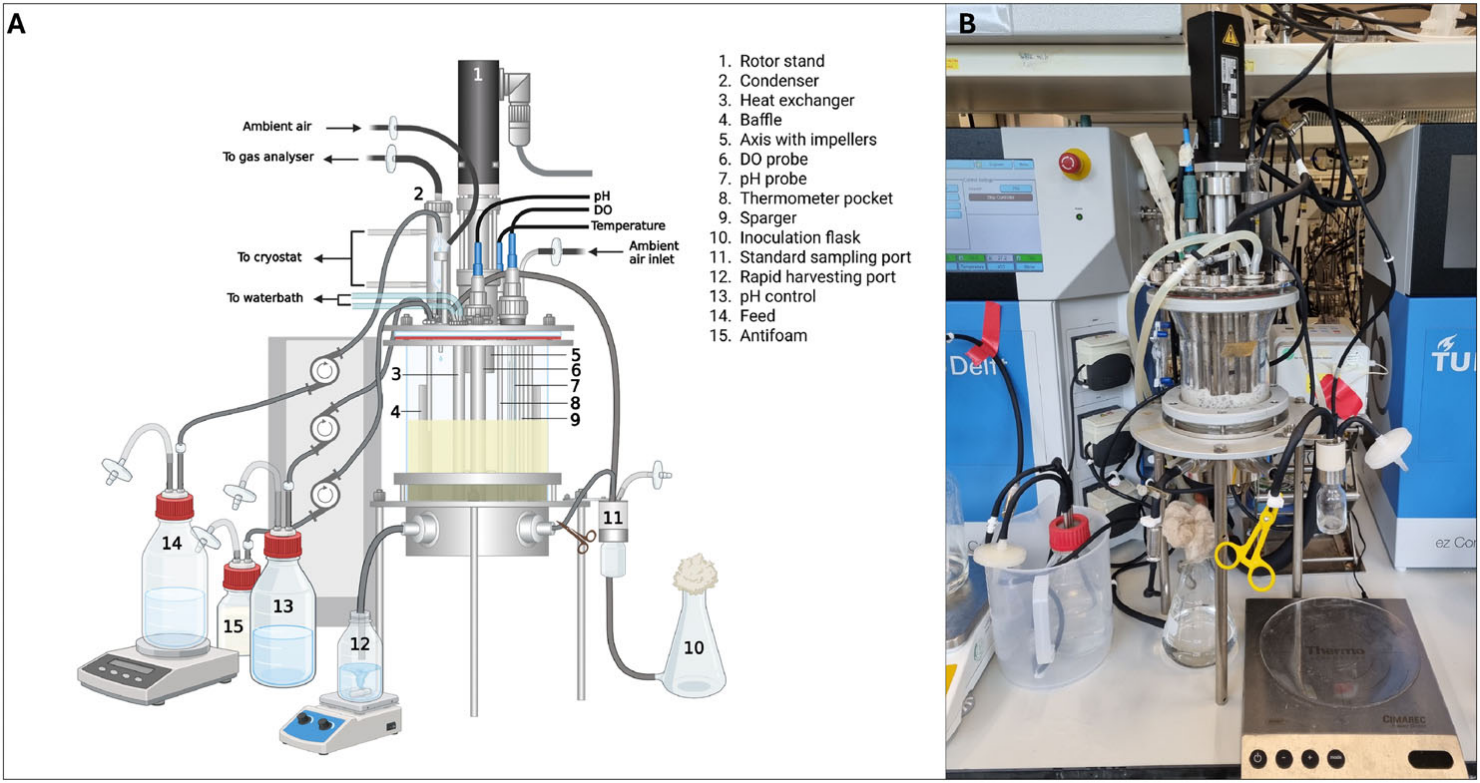
Schematic overview and photo of the fermentation and harvesting set‐up. (**A**) Schematic representation of the fed‐batch and rapid harvesting setup. Created using https://BioRender.com. (**B**) Picture of the fed‐batch setup at the end of the fermentation.

2 Calibrate the pump for fed‐batch medium inflow at a flow range of 0.02‐0.5 ml/min.
3 Prepare batch medium without vitamins and glucose and add 350 ml of batch salt base to the fermentor.
4 Calibrate the pH probe (Fig. 2, number 7).
5 Sterilize the fermentor at 121°C and let it cool down.
6 Calibrate the DO probe (Fig. 2, number 6).
7 Attach the base (or acid; Fig. 2, number 13) and antifoam bottles (Schott bottles with screw caps with two or three inlets/outlets; Fig. 2, number 15) to the bioreactor, and ensure tubing is already filled with fluid before the fermentation starts.
  Acid may be used for pH control of cultures that alkalinize the medium during growth.
8 Set temperature control to 30°C and pH control to 5.

#### Initial batch fermentation

9 Set the gas inflow to 0.5 liters/min and the stirrer speed to 500 rpm.
10 Add 100 ml glucose solution (for a glucose starting concentration of 75 mM), 1.5 ml filter‐sterilized vitamin solution, and 1 ml *S. cerevisiae* CEN.PK113‐7D glycerol stock to the inoculation flask (Fig. 2, number 10), and then add the flask contents to the fermentor.
11 Flush the inoculation flask by adding 50 ml sterilized demi water and transferring it to the fermentor.
  The starting volume in the bioreactor is ∼500 ml.
12 Set the pressure on the bioreactor to 0.3 bar.
13 If excessive foaming occurs, add a few drops of antifoam C solution by briefly switching on the pump.
14 Sample at regular time intervals for extracellular metabolites and dry weight measurements using a standard sampling port (Fig. 2, number 11; see Support Protocol 1).
15 Continuously monitor the CO_2_ profile over time using a gas analyzer. Arrest of CO_2_ and biomass production indicates that all carbon is depleted in the batch phase and that the fed‐batch phase can be started (Fig. 3).

**Figure 3:**
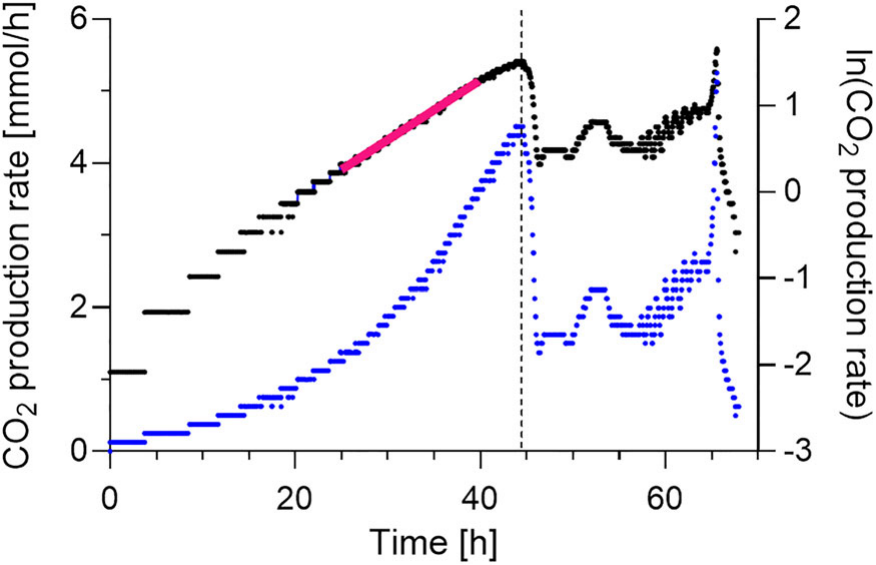
CO_2_ production profile during the batch phase. The CO_2_ production rate (in millimoles per hour) is depicted in blue (left *y‐*axis) and the natural logarithm (ln) of the CO_2_ production rate is depicted in black (right *y*‐axis). The trendline through ln(CO_2_ production rate) gives an approximation of the maximum growth rate. For this culture, using timepoints from 20 hr to 40 hr (purple line) gives an estimated growth rate of 0.0692 h^−1^ (linear regression equation *y* = 0.0692*x* − 1.4735, with *R* ^2^ = 0.995).

#### Fed‐batch fermentation

16 Calculate the fed‐batch feeding profile based on the final biomass concentration after the batch fermentation following Equations (1) and (2) (Heijnen, 2009; Hensing et al., 1994).
(1) $$F_{s}=f\cdot e^{\mu \cdot t}$$
(2) $$f=\left(\frac{\mu }{Y^{\frac{x}{s}}}+m_{s}\right)\cdot \left(\frac{c_{x\cdot 0}\cdot V_{0}}{C_{s,\mathrm{in}}}\right)$$
  With:
*F*_s_ [L h^−1^] = volumetric substrate feed rate
*f* [L h^−1^] = feed factor
*t* [h] = time
  µ [h^−1^] = growth rate
*Y*_x/s_ [g_s_ g_x_^−1^] = yield of biomass (x) on substrate (s), organism dependent
*m*_s_ [g_s_ g_x_^−1^ h^−1^] = maintenance requirements, organism dependent
*c_x_*_,0_ [g_x_ liter^−1^] = biomass concentration in the bioreactor at the end of the batch phase
*V*_0_ [L] = volume in the bioreactor at the end of the batch phase
*c*_s, in_ [*g*_s_ liter^−1^] = substrate concentration in the feed
    The Y_x/s_ and m_s_ values are dependent on the organism and the cultivation conditions. For S. cerevisiae grown on glucose, Y_x/s_ and m_s_ are 0.5 g_s_ g_x_^−1^ (van Hoek et al., 2000) and 0.0072 g_s_ g_x_^−1^ hr^−1^ (Vos et al., 2016), respectively. These values can be found in the literature for various model organisms or can be experimentally determined using chemostat cultures. In the fed‐batch phase, the specific growth rate, µ, is set by the feeding rate. If the specific growth rate is set to a value above the organism's maximum growth rate, the feeding rate will be too high and glucose will be supplied in excess, resulting in respiro‐fermentative metabolism. It is therefore important to determine the maximum specific growth rate of the organism under the chosen cultivation conditions, for instance by measuring biomass concentration (optical density) during the batch phase or by performing a separate shake flask experiment. For S. cerevisiae under the given cultivation conditions, the maximum growth rate measured in batch culture is ∼0.07 hr^−1^ (Fig. 3) and the growth rate for the fed‐batch phase was set at 0.05 hr^−1^. The initial volume in the bioreactor at the start of the fed‐batch fermentation is 0.5 liters and the substrate concentration in the feed is 116.41 gL^−1^. Depending on the total working volume of the bioreactor, sparging and gas inflow capacity, and feeding pump capacity, the initial volume in the bioreactor and substrate concentration of the feed might need to be adjusted. Finally, to calculate the feeding profile, the biomass concentration must be accurately measured (see Support Protocol 1).
17 Start the fed‐batch fermentation by switching on the feeding pump with fed‐batch medium at the calculated rate.
  Verify the unit of the flow rate required by the pump controller—whether it is specified in milliliters per minute or liters per hour. Additionally, confirm whether the exponential factor—e^µt^ (Equation 1)—is applied automatically by the software, or if it needs to be manually adjusted within the program.
18 Take samples for determination of extracellular metabolites and determination of dry weight (see Support Protocol 1) at regular intervals during the fed‐batch fermentation to monitor progress and to check whether the biomass concentration is in line with the expected profile (see Supplemental File 1 for the simulated biomass profile during the fed‐batch process; Fig. 4). For extracellular metabolites, spin down samples, transfer supernatant to an HPLC vial, and analyze the substrate (here, glucose) and product (ethanol and organic acids for yeast) concentrations by standard gas or liquid chromatograph methods.
  We recommend taking at least five samples during the fed‐batch phase to ensure that the feeding profile has been set correctly.

**Figure 4:**
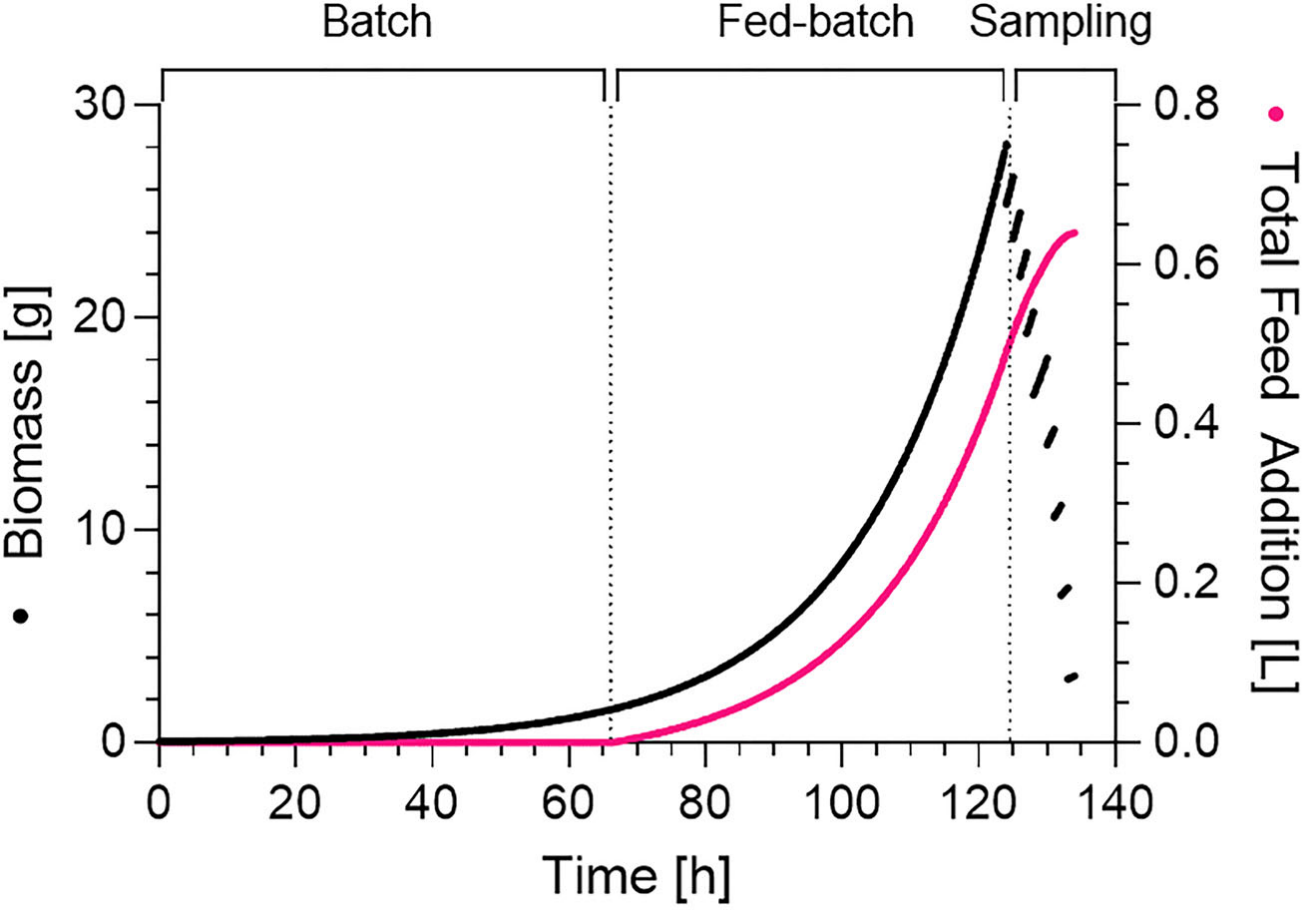
Simulated medium feeding and biomass profiles during the fed‐batch and harvesting phases. The cumulative volume of medium fed to the bioreactor (right axis) and corresponding biomass profile (left axis) were extrapolated from the dry weight determination at the end of the batch phase.

#### Harvesting from the bioreactor

The harvesting will mimic the harvesting phase that will be performed at the end of the real run with [^13^C]glucose and is a good rehearsal for the harvesting team. The harvesting time should be carefully chosen based on two factors. On one hand, it is important to harvest while medium is still being supplied to the culture to prevent carbon starvation and metabolite depletion. On the other hand, as little as possible of the [^13^C]glucose‐containing medium should be left unused. To prevent excess feeding of glucose during the harvesting phase, the feeding rate should be adjusted according to the broth volume remaining in the fermentation (see step 24).

To check when the medium will be exhausted, see cell 4E in Supplemental File 1 (the spreadsheet “FEDBATCH_SAMP”). The volume in the bioreactor after harvesting should be as close to zero as possible; see cell 58E in the same sheet.

19 When ready to begin harvesting, attach two 50‐ml rotors, prechilled to −40°C, to the centrifuges.
20 Immediately before harvesting, remove a Schott bottle containing 450 ml of 100% methanol from the −40°C freezer, weigh it rapidly, and record the weight.
  This step is important to monitor how much volume is removed from the fermentor.
21 Place the bottle under the rapid harvesting port (Fig. 2, number 12) on a stir plate and turn on the stirrer. Stir at a steady, medium speed so that the broth and methanol mix as quickly as possible while minimizing the risk of methanol splashing.
22 Sample ∼100 ml of broth directly into the Schott bottle with methanol. Use the volume indicator on the bottle at 550 ml as a guideline.
  The volume of broth harvested is limited by the capacity to process samples rapidly: for instance, the number of tubes that can be spun down simultaneously, the number of people available for fast handling of the samples, and the capacity of the −40°C freezer. If the centrifugation capacity in the lab is limited to six 50‐ml Greiner tubes per round, the remaining six 50‐ml Greiner tubes can be stored in a −40°C freezer to keep the samples from heating up too much. A critical requirement for proper quenching is that the harvesting always be performed in an approximately 1:5 broth‐to‐methanol ratio (Mashego et al., 2004).
23 Weigh the bottle containing the broth/methanol mixture and calculate the sampled volume.
24 Adjust the feed rate according to the amount of harvested broth to keep the growth rate constant. Record the recorded weight of the broth in cell O17 for the first sample, O22 for the second sample, and so on, using Supplemental File 1 (spreadsheet “FEDBATCH_SAMP”). Based on the sampling volume, the feed rate is automatically calculated in the spreadsheet; see cell J18 for the adjusted feed rate in liters per hour and cell K18 for the feed rate in milliliters per minute.
  For a detailed explanation of the calculations, see Supplemental File 2. Briefly, first calculate the new volume in the bioreactor, based on V = V0 + V_feed − V_sampling. In this formula, V_feed is obtained by integration of equation 2; ΔV_feed = ∫ Fs = (f/mu)*exp(mu*t) − (f/mu), and V_sampling is obtained by weighing the sample. Next, adjust the total amount of biomass in the reactor after harvesting (M_X_#), which can be determined based on the volume of the sample (ΔV_sample) as a fraction of the total volume (V); M_X_# = M_X * (1 − (ΔV_sample/V)). Based on the adjusted volume and biomass concentration, the adjusted feed rate can be calculated according to Equation 2.
  Because of the discrepancy between exponential increase of the total volume in the reactor and the total amount of biomass in the reactor, it is important to calculate the total amount of biomass rather than the biomass concentration for determining the feeding profile.
25 Proceed to Basic Protocol 2 for metabolite extraction.
26 Repeat steps 19‐25 until the entire bioreactor is empty.

*S. cerevisiae* cultures with fully respiratory metabolism on glucose have a respiratory quotient (RQ, ratio of the carbon dioxide production rate to the oxygen consumption rate) of ∼1.06. Respiro‐fermentative metabolism will lead to increased RQ. Online monitoring of the culture RQ therefore makes it possible to assess whether the feed rate is set in the correct range. If the RQ exceeds 1.2, it is advisable to adjust the feed rate accordingly. Figure 5 depicts the RQ profile during batch and fed‐batch culture of *S. cerevisiae*. The RQ is high (>2) during the glucose consumption phase in batch, then decreases to ∼0.5 during the following ethanol and organic acid consumption phase in batch and subsequently stabilizes at ∼1.1 in the fed‐batch phase.

**Figure 5:**
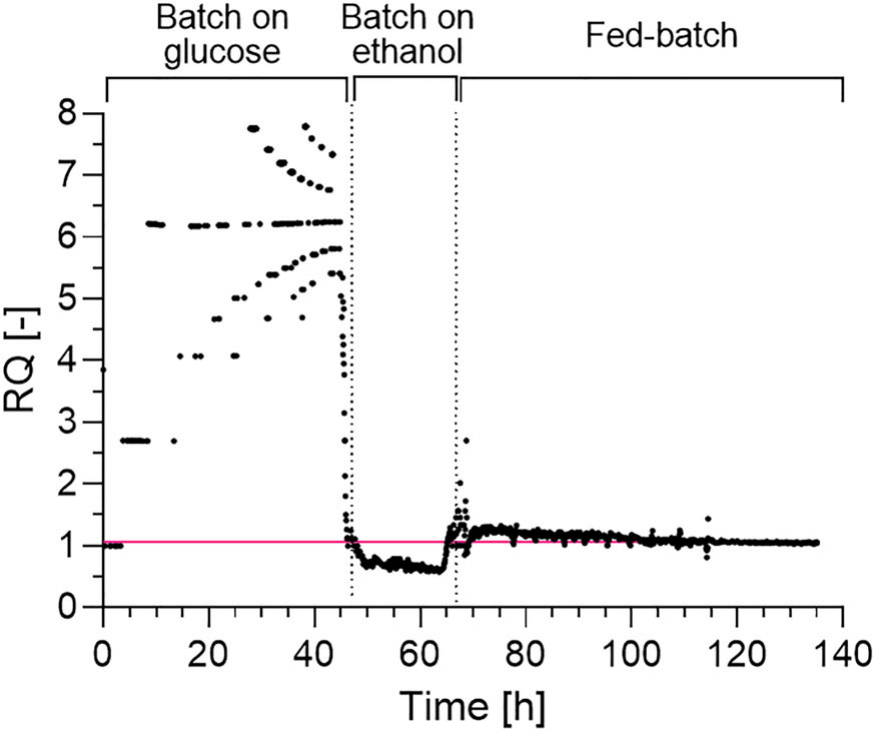
Respiratory quotient (RQ) profile during fermentation. The RQ, the ratio of the carbon dioxide production rate to the oxygen consumption rate, is used as proxy for the metabolic mode during fermentation. The pink line indicates fully respiratory metabolism, with an RQ of 1.06. The scattering of RQ values in the batch phase reflects the low biomass concentration in the bioreactor and resulting low CO_2_ production and O_2_ consumption rates that approach the detection limit of the gas analyzer.

## Support Protocol 1 DRY WEIGHT DETERMINATION

This protocol describes how to determine the dry weight of the culture, according to Postma et al. (1989). Always perform the dry weight determinations in duplicate.

### Materials

Nitrocellulose filters, 0.45‐µm pore size
Demineralized (demi) water
Yeast culture samples (Basic Protocol 1)

Oven, 80°C
Glass Petri dish
Flat‐tip tweezer
Precision balance (≥0.001 g)
Vacuum‐pump connected to a filtration device
10‐ml calibrated volumetric pipet
Microwave

1 Dry the nitrocellulose filters for a minimum of 8 hr at 80°C in an oven.
  The filters can be kept in the oven for prolonged periods of time.
2 Place the blue separation paper disk included in the filter box in a dry glass Petri dish. Collect the filter that will be used in determining dry weight, place it on the blue disk, and close the petri dish.
  Always handle the filter using a flat‐tip tweezer, avoid bringing the filter in contact with moist materials.
3 Weigh the dried filter, without the blue separation paper, on a precision balance, and wait until the signal stabilizes.
4 Switch on the vacuum pump connected to the filtration device and place the dried filter on the filtration device.
5 Wet the filter with ∼10 ml demi water.
6 Mix the culture sample, by shaking, and slowly pipet, using a calibrated volumetric pipet, exactly 10 ml of the culture sample onto the filter. For cultures with low biomass concentration (<0.3 g/liter), adjust the volume of sample (e.g., to 20 ml).
7 Carefully wash the filter with the cell broth with 20 ml demi water to remove salts. Avoid splattering.
8 Remove the filter from the filtration device using tweezers (be careful not to touch the cell material).
9 Place the blue separation disk on the microwave dish and place the filter on top of the disk to prevent the filter from sticking to the microwave dish.
10 Dry the filter in the microwave at low power settings: for example, 350 W for 20 min using defrost.
11 Immediately after drying, weigh the filter on the precision balance.
12 Calculate the dry weight in grams per liter by subtracting filter mass from filter mass with biomass. Deviation between duplicates should be <1%.

## Basic Protocol 2 QUENCHING AND WASHING OF CELL SAMPLES AND METABOLITE EXTRACTION

This protocol describes how to rapidly quench cellular metabolism by direct mixing of the broth into cold methanol, prevent contamination of cell extracts by metabolites present in the medium by cold centrifugation and washing, and extract the metabolites from the cells with boiling ethanol. After metabolite extraction, ethanol is evaporated, cells are resuspended in water, and the resulting metabolite extract is separated from cell material by centrifugation and filtration. The same protocol is used to produce cell extracts from the trial run (^12^C only, Basic Protocol 1) and the real run (^13^C only, Basic Protocol 5) and finally in quantifying metabolite concentrations in samples of interest using internal standard (^12^C extract with ^13^C‐labeled internal standard, Basic Protocol 8). For the latter, internal standard is spiked before the metabolite extraction using boiling ethanol at step 25. Upon successful completion of this protocol, the cell extract is aliquoted and stored at −80°C.

### Materials

Yeast bioreactor cultures (Basic Protocol 1)
Methanol (HPLC grade, >99.9%), −40°C
83% (v/v) methanol in demineralized (demi) water, −40°C
Internal standards (Basic Protocol 8), if used
75% (v/v) ethanol (>99.8%)
Milli‐Q‐purified water

Freezers, −40°C (for methanol storage) and −80°C (for sample storage)
Cryostat with cryofluid (70% [v/v] ethylene glycol), capable of reaching −40°C
1‐liter Schott bottles
5‐ and 20‐ml‐capacity bottle‐top dispensers
Water baths, 75°C and 95°C
50‐ml Falcon tubes (135)
Tabletop centrifuge (−19°C), with rotors for 15‐ and 50‐ml tubes, stored at −40°C
Waste bucket for methanol‐contaminated waste (plastic and liquid)
Vortex mixer
Standard 14‐mm‐diameter marbles
Bucket with ice, sized to fit 12 × 50‐ml Falcon tubes comfortably
Benchtop centrifugal vacuum concentrator (SpeedVac): e.g., Labconco CentriVap or equivalent, with vacuum pump and cold trap, capable of evaporating ethanol and equipped with a rotor for 15‐ml conical tubes
15‐ml sampling tubes sized to fit the SpeedVac inlets (135 tubes)
Syringes
0.2‐µm‐pore‐size PVDF syringe filters
Nitrile gloves

#### Harvesting from the bioreactor

1 Turn on the cryostat(s) and centrifuge(s) and allow them to reach the desired temperature.
2 Place four bottles filled with 83% (v/v) methanol solution in the cryostat and attach a bottle‐top dispenser to one of the bottles.
3 Turn on the two water baths to 75°C and 95°C.
4 Put twelve 15‐ml Falcon tubes containing 75% (v/v) ethanol, or a Schott bottle filled with 75% ethanol and equipped with a bottle‐top dispenser, into the 75°C water bath.
  Always keep the samples on ice, at least until the boiling ethanol step (step 25).
5 Harvest the broth in cold methanol as described in Basic Protocol 1, steps 20‐23.

#### Removal of extracellular metabolites

6 Immediately after harvesting, transfer the quenched broth to the twelve 50‐ml Falcon tubes (∼48 ml per tube) by pouring.
  Pouring of the broth‐methanol solution should be done in a fume hood for safety reasons.
7 Centrifuge 5 min at 2500 × *g*, −19°C.
8 Decant the supernatant into the methanol waste vessel.
9 Add 20 ml prechilled (−40°C) 83% (v/v) methanol to all pellets using the bottle‐top dispenser.
10 Resuspend the pellets by vortexing.
11 Centrifuge 5 min at 2500 × *g*, −19°C.
12 Remove the rotor for 50‐ml tubes and replace it with a rotor for 15‐ml tubes; place the other rotor back in –40°C freezer.
13 Adjust the bottle‐top dispenser on the 83% (v/v) methanol to 3 ml.
14 Decant the supernatant into the methanol waste vessel.
15 Add 3 ml prechilled (−40°C) 83% (v/v) methanol to all pellets using the bottle‐top dispenser.
16 Resuspend the pellets by vortexing.
17 Pour the liquid into the twelve 15‐ml Falcon tubes.
18 Wash the 50‐ml Falcon tubes with 3 ml each of prechilled (−40°C) 83% (v/v) methanol using the bottle‐top dispenser.
19 Pour the liquid into the twelve 15‐ml sampling tubes (do not leave any biomass behind).
20 Centrifuge 5 min at 2500 × *g*, −19°C.
21 Decant the supernatant into a waste vessel.
*IMPORTANT NOTE*: If you process samples for which you want to use internal standards for quantification of intracellular metabolites, internal standards should be added at this step (see Basic Protocol 8).

#### Metabolite extraction

22 Add 5 ml of prewarmed (75°C) 75% (v/v) ethanol.
23 Resuspend the pellet by vortexing.
24 Close the sampling tubes by placing a marble on top of each tube.
  Sampling tubes should not be closed using the original caps because the caps will pop open.
25 Boil the samples 6 min by placing them in the 95°C water bath.
26 Place the tubes on ice.
27 *Optional*: At this stage, samples may be stored at −80°C for up to 4 days.
28 Evaporate the ethanol in the SpeedVac following the manufacturer's instructions.
  This typically takes ∼12 hr.
  It is recommended that the program be executed at room temperature. To prevent breakdown of metabolites, do not heat.

#### Filtration and analysis

29 Resuspend the pellet in Milli‐Q‐treated water. Adjust the volume of water to achieve 10 mg biomass per 100 µl of final volume.
  The amount of biomass in the sample can be determined based on the dry weight measurement taken at the end of the fed‐batch stage during the trial run. 10 mg biomass per 100 µl final volume was chosen based on previous research that found that this concentration should be sufficient to measure central carbon metabolism intermediates (Canelas et al., 2009; Mashego et al., 2004; Wasylenko & Stephanopoulos, 2015).
30 Centrifuge the resuspended cell extracts to remove cell debris for 5 min at 17,000 × *g*, 4°C. Recover the supernatant containing the cell extract and discard the pellets.
31 Centrifuge the recovered supernatant again for 5 min at 17,000 × *g*, 4°C, to ensure removal of all cell debris, considering the high biomass concentration.
32 *IMPORTANT*: When preparing internal standards, pool all resuspended cell extracts from all samples in a single flask to obtain a homogenous batch of ^13^C‐labeled yeast extract.
33 Filter the (pooled) cell extract using 0.2‐µm‐pore‐size syringe filters.
34 Depending on the type of analysis that will be performed, proceed as follows:
  a. For analysis of the ^12^C yeast extract from the trial run, proceed to Basic Protocols 3 and 4.
  b. For characterization of the ^13^C‐labeled yeast extract, continue to Basic Protocols 6 and 7.
  c. For preparation of the internal standard only, divide the ^13^C‐labeled yeast extract in screw‐cap vials (1‐2 ml extract per vial) and store at −80°C.
*IMPORTANT NOTE*: If metabolites of interest are not present or are insufficiently abundant in the ^13^C yeast extract, spike the internal standard with commercial ^13^C‐labeled compounds at this stage, before dividing between vials.
  d. For quantification of metabolites in a sample of interest using internal standard, continue to Basic Protocol 8.

## Basic Protocol 3 IDENTIFICATION OF INTRACELLULAR METABOLITES USING LC‐MS

Metabolites that are polar and contain phosphate groups, such as nucleotides, are best separated by LC using a porous graphitic carbon stationary phase (PGC). This protocol describes how to prepare samples to separate these compounds using PGC‐LC and how to identify them using high‐resolution MS with metabolites involved in the NAD(P)^+^ biosynthesis in *S. cerevisiae* as example.

### Materials

Yeast cell extracts (Basic Protocol 5)
^12^C yeast cell extract (Basic Protocols 1 and 2)
Optima LC‐MS‐grade H_2_O (Fisher Chemical)
^12^C commercial compounds of interest, highest purity
Pierce LTQ ESI negative‐ion calibration solution (Thermo Fisher Scientific)
10 mM ammonium bicarbonate in 60% acetonitrile (see recipe)
Optima LC‐MS‐grade acetonitrile (Fisher Chemical)
Mobile phase A: 50 mM ammonium bicarbonate in water (see recipe)
Mobile phase B: 100% Optima LC‐MS‐grade acetonitrile (Fisher Chemical; keep a 50‐ml aliquot at 4°C)
Seal wash: 10% acetonitrile/10 mM ammonium bicarbonate in water (see recipe)
Strong wash: 10% acetonitrile/10 mM ammonium bicarbonate in water (see recipe)
Weak wash: 50 mM ammonium bicarbonate in water (see recipe)

SPE column plate: HyperSep 96 Hypercarb 25 mg/1 ml Plate (Thermo Scientific)
Chromatography column: Hypercarb Porous Graphitic Carbon HPLC Column, 100 × 1 mm, Thermo Scientific, Part no. 35005‐101030, particle size 5 µm
2‐ml Square Collection Plate (part no. 186002482, Waters)
Collecting tubes, strips of 8 tubes (cat. no. SIMPT110‐15, Avantor)
1.5‐ml microcentrifuge tubes
SpeedVac
LC‐MS vials: polypropylene 12 × 32‐mm Screw Neck Vial, with Cap and Preslit PTFE/Silicone Septum, 300 µl Volume (Waters)
Acquity Ultra Performance Liquid Chromatograph (Waters)
Q Exactive Focus Hybrid Quadrupole‐Orbitra Mass Spectrometer (Thermo Scientific)
500‐µl glass syringe for HPLC instruments (Thermo Scientific)
Analysis software (e.g., Xcalibur 4.1, Thermo Scientific)
Microcentrifuge, speed 6000 rpm (Roth) or any other centrifuge for Eppendorf (microcentrifuge) tubes

*CAUTION*: Electrical grounding of the porous graphitic carbon column is required to prevent column polarization caused by the electrospray voltage (Pabst & Altmann, 2008).

*CAUTION*: Retention time (RT) instability is often observed when using porous graphitic carbon as the chromatographic separation phase. Washing steps and regular backflushing may solve the problem (Bapiro et al., 2016). In this study, a mobile phase A, containing 50 mM ammonium bicarbonate, was used to limit the RT variability. Nevertheless, RT instability should be kept in mind and should be evaluated when performing the analyses. If necessary, inject the standards again separately or as a mixed solution before the analyses of the yeast cell extract.

*NOTE*: Whenever water is mentioned, this refers to Optima LC‐MS‐grade water.

#### Cleaning up the cell extracts using solid‐phase extraction (SPE)

1 Wash and concentrate the cell extracts from Basic Protocol 2 using a HyperSep PGC column plate, as follows:
  • Place a waste tray under the SPE columns.
  • Condition step: Add 600 µl of 10 mM ammonium bicarbonate in 60% acetonitrile to the SPE columns.
  • Equilibration step: Add 600 µl water to the SPE columns. Repeat.
  • Loading step: Pipet the cell extract samples onto the SPE columns.
  • Washing step: Add 1000 µl water to the SPE columns.
  • Remove the waste tray and place the sample collection plate with new collecting tubes underneath the SPE columns.
  • Elute the samples by adding 200‐µl aliquots of 10 mM ammonium bicarbonate in 60% acetonitrile to the SPE columns and repeating.
2 Transfer the samples from the collecting cups to 1.5‐ml microcentrifuge tubes.
3 Dry the collected samples using the SpeedVac concentrator on the “no temperature” setting. Leave the lid of the microcentrifuge tubes open.
  This step usually takes 3‐4 hr.
4 *Optional*: Store the dried samples at −80°C until analysis.
5 Dissolve the samples in 20 µl of cold water each.
6 Vortex the samples for a couple of minutes.
7 Gather all liquid at the bottom of the microcentrifuge tubes by briefly centrifuging the samples (∼30 sec) using a microcentrifuge.
  After centrifugation, you may notice a small black pellet in the tube. During elution from the PGC SPE columns, some of the graphite carbon might enter some samples. Avoid pipetting this black pellet into your LC‐MS vial—only take the clear supernatant.
8 Transfer each supernatant to an LC‐MS vial. The samples can now be analyzed by LC‐MS.

#### Identifying metabolites using LC‐MS

9 Prepare separate solutions of commercial compounds of interest dissolved in water, using the concentrations shown in Table 1.

**Table 1:** Concentrations of Commercial Compounds of Interest in the Calibration Solutions

Compound	Concentration (mM)	Compound	Concentration (mM)
PRPP	13	NR	27
ATP	13	NAM	27
ADP	27	NA	20
NADPH	13	Trp	16
NADP	27	QA	1
NADH	27	NMN	27
NAD	27	NaMN	13
NaAD	27		

PRPP, 5‐phosphoribosyl‐1‐pyrophosphate; ATP, adenosine triphosphate; ADP, adenosine diphosphate; NAD(P)(H), nicotinamide adenine dinucleotide (phosphate); NaAD, nicotinic acid adenine dinucleotide; NR, nicotinamide riboside; NAM, nicotinamide; NA, nicotinic acid; Trp, tryptophan; QA, quinolic acid; NMN, nicotinamide mononucleotide; NaMN, nicotinic acid mononucleotide.

Concentrations were rounded to whole number.

10 Perform a mass calibration in negative‐ion mode using 500 µl Pierce LTQ ESI negative‐ion calibration solution.
11 Create a full MS method using the following settings:
  • Method duration: 31 min
  • Polarity: Negative
  • Resolution: 35,000
  • No. scan ranges: 1
  • Scan range: 75‐1000 *m*/*z*
  • Automatic gain control (AGC) target: 1.00E+06
  • Maximum injection time (IT): 100 ms
  • Microscans: 2
  • Spectrum data type: Profile
12 Carefully purge the HPLC system with 50% mobile phase A (50 mM ammonium bicarbonate in Optima LC‐MS‐grade H_2_O) and 50% mobile phase B (Optima LC‐MS‐grade acetonitrile) for 10 min. Equilibrate the mounted and grounded PGC column overnight in the starting conditions.
13 Create an HPLC method using the following settings:
  • Flow rate: 0.100 ml/min
  • Set the gradient as described in Table 2
  • Seal wash: 2.5 min

**Table 2:** Applied Gradient for Separation of Metabolites by Liquid Chromatography

Time (min)	%A	%B	Curve
Initial	97.5	2.5	Initial
2.0	97.5	2.5	6
20.0	65	35	6
26.0	40	60	6
27.0	97.5	2.5	6
31.0	97.5	2.5	6

Mobile phase A consists of 50 mM ammonium bicarbonate in Optima LC‐MS‐grade water, and mobile phase B consists of Optima LC‐MS‐grade acetonitrile. Curve 6 denotes linearity.

14 When creating the sample list, set the injection volume to 1 µl and run two blanks, consisting of Optima LC‐MS‐grade H_2_O, in between samples.
15 Place the solutions to analyze (cell extract or commercial compound) in the autosampler and start the run.
16 Analyze the mass spectra.
  We use Xcalibur 4.1 (Thermo Scientific), but any other suitable software can also be used.
  This example focuses on the identification of NAD^+^‐related metabolites, but many other metabolites can be identified if commercial compounds are available for calibration.
  Extracted ion chromatograms were created of the metabolites of interest using a 10‐ppm error range, after which the area of the peaks was integrated.
*IMPORTANT NOTE*: This identification step determines which metabolites can be measured in the ^12^C yeast cell extract, and hence in the internal standard. Some metabolites might not be measurable due to several factors, such as concentration below their detection limit or chemical instability. For instance, nicotinic acid, although stable, is found at concentrations close to the detection limit in yeast cell extracts. The yeast‐based internal standard therefore cannot be used to quantify nicotinic acid. This caveat can be mitigated by addition of commercial, ^13^C‐labeled nicotinic acid to the custom‐made internal standard.
17 Determine the retention time of each standard. The *m*/*z* of each unlabeled metabolite (negatively charged) is provided in Table 3.

**Table 3:** Selection of Metabolites in NAD^+^ and ATP Metabolism Detectable by LC‐MS and Masses of the Corresponding ^12^C and ^13^C Variants

Compound	Monoisotopic mass	Chemical formula [M‐H]^−^	^12^C *m*/*z*	^13^C *m*/*z*
PRPP	389.9518	(C_5_H_12_O_14_P_3_)^−^	388.9445	393.9613
ATP	506.9958	(C_10_H_15_N_5_O_13_P_3_)^−^	505.9885	516.0220
ADP	427.0294	(C_10_H_14_N_5_O_10_P_2_)^−^	426.0221	436.0557
NADPH	745.0911	(C_21_H_29_N_7_O_17_P_3_)^−^	744.0838	765.1543
NADP	744.0833	(C_21_H_27_N_7_O_17_P_3_)^−^	742.0682	763.1386
NADH	665.1248	(C_21_H_28_N_7_O_14_P_2_)^−^	664.1175	685.1880
NAD	664.1164	(C_21_H_26_N_7_O_14_P_2_)^−^	662.1019	683.1723
NaAD	665.1004	(C_21_H_25_N_6_O_15_P_2_)^−^	663.0859	684.1563
NR	255.0976	(C_11_H_13_N_2_O_5_)^−^	253.0830	264.1199
NAM	122.0480	(C_6_H_5_N_2_O)^−^	121.0407	127.0609
NA	123.0320	(C_6_H_4_NO_2_)^−^	122.0248	128.0449
Trp	204.0899	(C_11_H_11_N_2_O_2_)^−^	203.0826	214.1195
QA	167.0219	(C_7_H_4_NO_4_)^−^	166.0146	173.0381
NMN	334.0566	(C_11_H_14_N_2_O_8_P)^−^	333.0493	344.0862
NMN fragment	212.0086	(C_5_H_8_O_7_P)^−^	211.0008	216.0181
NaMN	335.0406	(C_11_H_13_NO_9_P)^−^	334.0333	345.0702

18 Identify metabolites of interest in the cell extract by comparing mass and retention time with the values obtained with the unlabeled commercial compounds.
  In our analysis, the commercial compounds 5‐phosphoribosyl‐1‐pyrophosphate (PRPP) and quinolic acid (QA) could be detected, but they were not well retained on the PGC column and eluted almost immediately. In the ^12^C yeast cell extract, concentrations of PRPP and QA were below their detection limits and thus they could not be identified by LC‐MS.

## Basic Protocol 4 IDENTIFICATION OF INTRACELLULAR METABOLITES USING GC‐MS

Metabolites that can become volatile and are thermally stable are best separated by gas chromatography. Metabolites of central carbon metabolism (e.g., glycolysis, TCA cycle, and pentose phosphate pathway) and amino acids are such metabolites; however, they require chemical modification to meet the requirements of GC‐MS‐based separation and identification. Cell extracts are therefore freeze‐dried and derivatized before GC‐MS analysis. Glycolytic intermediates undergo oximation and silylation to form trimethylsilyl‐methoxamine hydrochloride (TMS‐MOX) derivatives. Amino acids are silylated to obtain *tert*‐butyldimethylsilyl chloride (TBDMS) derivatives. Once the metabolites of interest have been successfully identified in the ^12^C yeast cell extract, ^13^C‐labeled yeast extract can be produced using Basic Protocol 5.

### Materials

Compounds of interest, p.a. or higher purity
Ultrapure water
Yeast cell extracts (Basic Protocol 2)
NaCl, 99% pure
*N*‐Trimethylsilyl‐*N‐*methyl trifluoroacetamide (MSTFA)
Trimethylchlorosilane (TMCS; BGB Analytik)
Methoxyamine hydrochloride (MOX)
Pyridine, anhydrous, HPLC grade
Acetonitrile, HPLC grade
Alkane standard: saturated C7‐C30, 1000 µg/ml in hexane
Ethyl acetate, HPLC grade
Hexane, HPLC grade
Isooctane, HPLC grade
*N‐*Methyl*‐N‐(tert‐*butyldimethylsilyl)trifluoroacetamide (MTBSTFA) containing 1% *tert*‐butyldimethylchlorosilane

Pipets
Deactivated (silanized) 2‐ml clear glass vial (Agilent)
Short‐thread screw caps (transparent) with PTFE (Teflon) septa only (BGB Analytik)
Vacuum pump
Freeze dryer
Short‐thread screw caps (blue) with PTFE/silicone/PTFE septa (Agilent)
Freezer, −80°C
Fume hood
Nitrile gloves
Heating module, 70°C
1‐ml snap‐top shell vials, 8 mm, clear (Agilent)
Centrifuge
Vial inserts, pulled point glass (Agilent)
Helium source
GC system: 7890 A (Agilent Technologies) or equivalent
MS system: Triple Quad 7000 (Agilent Technologies) or equivalent
Zebron 50 ZB‐capillary column, 30 m × 250‐µm internal diameter, 0.25‐µm film (Phenomenex)
Straight glass liner, Ultra Inert, single taper with glass wool (Agilent)
10‐µl gas‐tight syringe with Teflon‐tipped plunger (Agilent)

*IMPORTANT*: For GC vials, silicone rubber should be avoided because of possible sample contamination and reactivity with the derivatization reagents.

#### Preparation of calibration solutions with unlabeled commercial compounds

1 Prepare stock solutions with commercial ^12^C compounds of interest in ultrapure water.
  During analysis, the concentrations of commercial compounds should be in the same range as expected in the sample to analyze. A typical commercial calibration solution has concentrations between 0.25 µM and 100 µM when optimized for S. cerevisiae; however, depending on the microorganism of interest, this concentration range may need to be adjusted. Use the information provided in the certificate of analysis from chemical providers for preparation of stock solutions.
2 From these stock solutions, prepare a calibration mix containing all commercial compounds (see Basic Protocol 7 for an example).
3 Prepare aliquots from this calibration mix and store at −80°C.
4 For preparation of a calibration range with these commercial compounds, see Basic Protocol 7.

#### Lyophilization of the cell extracts

5 Transfer 100 µl of cell extract or commercial compound to a silanized glass vial.
6 For amino acid analysis (but not for TCA and glycolysis metabolites), add 30 µl of 100 mg/ml NaCl in ultrapure water to each glass vial.
7 Close the vials with the caps containing white Teflon septa, and puncture two holes in each septum with a needle.
8 Freeze for at least 20 min at −80°C.
  Freezing samples at a 45° angle creates more surface area for lyophilization.
9 Place the frozen samples in a freeze‐dryer chamber.
10 Start the vacuum pump.
11 Place the samples in the freeze‐dryer overnight.
12 After freeze‐drying, re‐cap the vials with blue caps with PTFE/silicone/PTFE septa.
13 Store samples at −80°C until derivatization.

#### Derivatization of metabolites

*CAUTION*: Work in the fume hood and wear nitrile gloves during the derivatization.

##### For derivatization of central carbon metabolites (glycolysis, pentose phosphate pathway, and TCA metabolites)

14 Switch on the heating block to 70°C.
15 Prepare a MSTFA silylation reagent mix: Mix 1 ml of MSTFA with 50 µl TMCS. Use p.a. or higher purity.
  Derivatizing reagents should be stored in a desiccator in a 4°C refrigerator.
16 Prepare a solution of 20 mg/ml MOX in anhydrous pyridine. Vortex and preferably heat for 1 min in the heating block to let MOX completely dissolve (prepare freshly every day).
17 Add 50 µl of the MOX solution to each GC vial.
18 Incubate cell extracts or commercial compounds for 50 min at 70°C in the heating block.
19 Remove vials from the heating block and let them cool to room temperature.
20 Add 80 µl of the MSTFA silylation reagent mix to each vial.
21 Incubate the vials for 50 min at 70°C in the heating block.
22 Remove vials from the heating block and let them cool to room temperature.
23 Transfer samples from vials to glass centrifuge tubes (1‐ml shell vials).
24 Centrifuge 1 min at 10,000 × *g*, 4°C.
25 Transfer 90 µl of each supernatant from the centrifuge tube into a glass insert and place it back in the original GC vial (remove air bubbles if needed).

###### For derivatization of amino acids

26 Switch on the heating block (70°C).
27 Add 75 µl acetonitrile and 75 µl MTBSTFA containing 1% *tert*‐butyldimethylchlorosilane to each GC vial.
28 Incubate samples for 60 min at 70°C in the heating block.
29 Remove vials from the heating block and let them cool to room temperature.
30 Transfer samples into the glass centrifuge tubes (1‐ml shell vials).
31 Centrifuge 2 min at 10,000 × *g*, 4°C.
32 Transfer 90 µl of supernatant from the centrifuge tube into a glass insert and place it back in the original GC vial (remove air bubbles if needed).
  After these derivatization steps, the samples are ready for analysis by GC‐MS. The derivatized samples can be kept refrigerated at 4°C for up to 2 weeks.

#### Analyzing the derivatized metabolites from central carbon metabolism by GC‐MS

This analysis is performed according to Niedenführ et al. (2016) and Seifar et al. (2018), using a Zebron 50 ZB‐capillary column.

33 Set the carrier gas flow (helium 99.9990%) to 1 ml/min.
34 Program the GC oven temperature as follows: keep constant for 1 min at 70°C and then ramp up temperature at 10°C/min up to 220°C.
35 Set the temperature of the transfer line to 250°C.
36 Set the injection volume to 1 µl.
37 If using a multimode inlet (MMI; Agilent), use the following settings: split‐less mode for metabolites with low concentrations and split mode 1:5 for metabolites with high concentrations.
38 Set the MMI temperature: 70°C (with a split‐less time of 1 min if it is needed); after injection, increase the temperature to 220°C with a ramp of 720°C/min and hold for 5 min. Then increase the temperature to 300°C to clean the injector.
39 At the end of the run, backflush the column with five column volumes of helium at 220°C.
  For multiple injections from the same sample vial, replace the cap after each two injections, if possible.
  The liner in the GC might need to be replaced after different numbers of injections depending on the sample type. After ∼100 injections, cut off 10 cm of the column (injection side) and at the same time replace the liner and inlet septum (gas flow needs to be modified accordingly using retention‐time locking [RTL] to allow reproducible retention times).
  Run the alkane standard mix (20 µg/ml saturated alkane C7‐C30 in hexane) for RTL and system check about every ten samples.
40 Set the MS transfer line to 250°C.
41 Keep the MS source at 230°C.
42 Set the helium gas flow of the collision cell to 2.25 ml/min.
43 Set the N_2_ collision gas flow to 1.5 ml/min.
44 Use electron ionization operated at 70 eV.
45 Set the MS instrument to operate in SRM mode.
46 Set the mass resolution to 0.7 mass unit for both quadrupoles.
47 Run GC‐MS analysis.
  Before and after every injection, wash the needle using ethyl acetate and isooctane to prevent carryover between samples.
48 Mass Hunter analysis software (version 12.0/12.1; Agilent) can be used for data processing.
49 Integrate signals and qualify compounds using the commercial standard mix as a reference.
50 Identify all the compounds of interest in the unlabeled yeast extract sample based on the properties determined using the commercial compounds mix (Table 4).

**Table 4:** *m*/*z* Value, Number of Carbon Atoms, and Collision Energy of Some Central Carbon Metabolites of Interest

Compound	Precursor *m*/*z*	Original number of C atoms in precursor	Product *m*/*z*	Original number of C atoms in product	Collision energy
Fum	245.0	4	146.9	0e	10
Succ	247.0	4	147.1	0	5
Mal	335.0	4	146.9	0	15
aKG	304.0	5	146.9	0	5
G3P	445.1	3	299.2	0	15
GAP	400.1	3	299.0	0	10
Glc	319.1	4	129.1	3	10
Cit	465.1	6	146.9	0	35
iCit	465.1	6	146.9	0	35
2PG	459.1	3	299.1	0	15
DHAP	400.1	3	299.1	0	10
3PG	459.1	3	299.1	0	15
E4P	357.1	2	341.1	2	15
Rib5P	459.1	3	315.2	0	10
Ribu5P	357.1	2	147.1	0	35
Xyl5P	357.0	2	147.1	0	30
M6P	471.0	4	387.0	0	10
F6P	459.1	3	315.2	0	10
G6P	471.0	4	387.0	0	10
6PG	333.1	4	73.1	3	10
S7P	471.1	4	387.1	0	10
Tre	361.1	6	169.1	5	15
FBP	459.2	3	315.0	0	10
T6P	513.1	6	315.1	0	10

Fum, fumarate; Succ, succinate; Mal, malate; aKG, α‐ketoglutarate; G3P, glycerol‐3‐phosphate; GAP, glyceraldehyde‐3‐phosphate; Glc, glucose; Cit, citrate; iCit, isocitrate; 2PG, 2‐phosphoglycerate; 3PG, 3‐phsophoglycerate; DHAP, dihydroxyacetone phosphate; E4P, erythrose‐4‐phosphate; Rib5P, ribose‐5‐phosphate; Ribu5P, ribulose‐5‐phosphate; Xyl5P, xylulose‐5‐phosphate; M6P, mannose‐6‐phosphate; F6P, fructose‐6‐phosphate; G6P, glucose‐6‐phosphate; 6PG, 6‐phosphogluconate; S7P, sedoheptulose‐7‐phosphate; Tre, trehalose; FBP, fructose‐1,6‐bisphosphate; T6P, trehalose‐6‐phosphate.

#### Analyzing derivatized amino acids by GC‐MS (according to de Jonge et al., 2011)

51 Set the carrier gas flow (helium 99.9990%) to 1 ml/min.
52 Program the GC oven temperature as follows: keep constant for 1 min at 100°C and then ramp up by 10°C/min to 320°C.
53 Set the temperature of the transfer line to 250°C.
54 Set the injection volume to 1 µl.
55 If using a multimode inlet (MMI; Agilent), use the following settings: split‐less mode for metabolites with low concentration and in split mode 1:5 for metabolites with high concentrations.
56 Set the MMI temperature: 100°C (with a split‐less time of 1 min if it is needed); after injection, increase temperature to 220°C with a ramp of 720°C/min and hold for 5 min. Then, increase the temperature to 300°C to clean the injector.
57 At the end of the run, backflush the column with five column volumes of helium at 320°C.
  The comments for step 39 also apply to step 57.
58 Set the MS transfer line to 250°C.
59 Keep the MS source at 230°C and the quadrupole at 150°C.
60 Use electron ionization operated at 70 eV.
61 Set the MS to operate in SIM mode.
62 Set mass resolution to 1 mass unit throughout the mass range 1‐1050 amu.
63 Run GC‐MS analysis
64 Mass Hunter analysis software (version 12.0/12.1; Agilent) can be used for data processing.
65 Identify all the amino acids of interest in the unlabeled yeast cell extract based on the properties defined for the prepared commercial compounds mix (Table 5).

**Table 5:** *m*/*z* Value and Number of Carbon Atoms of Amino Acids

Compound	^12^C fragment *m*/*z*	Original number of C‐atoms in fragment	^13^C fragment *m*/*z*
Ala	260.1	3	263,1
Gly	246.1	2	248.1
Val	288.1	5	293.1
Leu	274.1	5	279.1
iLe	274.1	5	279.1
Urea	273.1	1	274.1
Pro	286.1	5	291.1
Ser	390.2	3	393.2
Thr	404.2	4	408.2
Meth	320.1	5	325.1
Asp	418.2	4	422.2
Phe	336.2	9	345.2
Cys	406.1	3	409.1
Glu	474.3	5	479.3
Lys	431.3	6	437.3
Asn	417.2	4	421.2
Gln	431.2	5	436.2
Tyr	466.3	9	475.3
His	440.3	6	446.3
Trp	375.2	11	386.2

Ala, alanine; Gly, glycine; Val, valine; Leu, leucine; iLe, isoleucine; Pro, proline; Ser, serine; Thr, threonine; Meth, methionine; Asp, aspartate; Phe, phenylalanine; Cys, cysteine; Glu, glutamate; Lys, lysine; Asn, asparagine; Gln, glutamine; Try, tyrosine; His, histidine; Trp, tryptophan.

## Basic Protocol 5 REAL RUN—FED‐BATCH

When the fed‐batch fermentation has reached (near) maximum biomass yield on glucose, meaning mainly respiratory metabolism for *S. cerevisiae*, and the ^12^C‐labeled yeast extract contains (most of) the metabolites of interest, you are ready for the “real” run. Afterward, sample processing can be performed as described in Basic Protocol 2, without any deviations.

To obtain a fully ^13^C‐labeled metabolite extract (the “real run”), perform Basic Protocol 1 with the following modifications to the indicated steps:

10. To prepare the batch, add the ^13^C glucose solution, and be aware that the ^13^C glucose solution requires a slightly different amount of glucose (see recipe in Reagents and Solutions).

14. Skip the sampling at regular time intervals.

The trial run will inform the real run, and no further samples should be taken during the fermentation to prevent loss of valuable ^13^C‐labeled yeast.

15. In monitoring the fermentation via gas analysis, it is essential to use a gas analyzer that can detect ^13^CO_2_ as previously described; however, even those gas analyzers will be less sensitive towards ^13^CO_2_, and as a result the signal will be weaker than in the trial run.

16. Skip step 16 and use the fed‐batch feeding profile calculated during the trial run.

18. Skip step 18.

No samples should be taken during the fed‐batch phase. If the RQ during the fed‐batch phase is too high according to CO_2_ and DO data, the fed‐batch profile should not be adjusted. During the real run, the data obtained is insufficient and not accurate enough to support an informed decision on adjusting the feed rate; therefore, the feeding profile determined during the trial run should be used.

19. Sample harvesting (steps 19‐26) can be done as described in Basic Protocol 1 with no adjustments. The ratio of methanol to broth and the timing of the sampling are kept the same. In Supplemental File 1 (spreadsheet “FEDBATCH_SAMP”), nine sampling points are suggested; however, the number of sampling points can be adjusted based on lab capacity. The use of nine sampling points was based on our lab's capacity, which was limited to one centrifuge capable of centrifuging six 50‐ml Greiner tubes at a time and sufficient −40°C freezer capacity for six 50‐ml Greiner tubes. In this situation, the leftover six tubes that could not be immediately centrifuged had to be kept in the –40°C freezer. The most important issue during sampling and subsequent sample processing is keeping the samples cold, i.e., quenched, at all times.

## Basic Protocol 6 LC‐MS CHARACTERIZATION OF THE INTERNAL STANDARD

Basic Protocol 6 describes the assessment of ^12^C contamination of the internal standard produced in Basic Protocol 5 using LC‐MS, the identification of the metabolites of interest, and the quantification of these metabolites in the internal standard using unlabeled commercial standards.

### Materials

See Basic Protocol 3

#### Evaluation of potential ^12^C contamination of metabolites in the internal standard

Although the culture medium has been optimized to minimize the occurrence of ^12^C in the internal standards, the purity of the ^13^C‐labeled yeast extract still needs to be evaluated. The number of ^12^C and ^13^C atoms in a metabolite will affect its mass, which can be detected by mass spectrometry. The internal standard should therefore be analyzed by LC‐MS as described in Basic Protocol 3.

Because the mass difference between a ^13^C and a ^12^C atom is 1.003354, the mass of the isotope variants can be calculated using equation 3:

(3) $$\mathrm{Isotope}\;\mathrm{mass}\;=\;\left(\mathrm{monoisotopic}\;\mathrm{mass}\;+\;N\right)\;*\;1.003354$$

where *N* represents the number of carbon atoms

For example, the ^12^C monoisotopic mass of ATP equals 506.99575, and ATP contains ten carbon atoms. Therefore, the mass of fully ^13^C‐labeled ATP becomes:

$$\left(506.99575\;+\;10\right)\;*\;1.003354\;=\;517.02929$$

The mass of ATP containing nine ^13^C atoms and one ^12^C atom becomes:

$$\left(506.99575+9\right)\;*\;1.003354\;=\;516.025936$$

Because the MS analyses are performed in negative‐ion mode, the mass of a proton must be subtracted to get the final *m*/*z* value, which can be used to create the extracted ion chromatograms (see Table 3).

1 Analyze the ^13^C yeast extract by following the instructions in Basic Protocol 3, steps 1‐16.
2 Create, for all isotope variants of each metabolite, an extracted ion chromatogram with a 10‐ppm error marge.
3 Integrate the peaks in Xcalibur or any other suitable software.
4 Calculate the percentage of isotope variants compared to the fully ^13^C‐labeled variant using Equation 4.
(4) $$\mathrm{Isotope}\;\mathrm{percentage}=\;\frac{\mathrm{Area}\;\mathrm{isotope}}{\mathrm{Area}\;\mathrm{fully}\;13C-\mathrm{labeled}\;\mathrm{metabolite}}*100\%\;$$
  Although full ^13^C labeling of all carbon atoms in all metabolites is difficult to attain and is not required for the internal standards, the absence of monoisotopic ^12^C compounds is a prerequisite to avoid interference between the internal standard and the unlabeled samples being analyzed. For instance, the internal standard produced with this protocol contained metabolites with one ^12^C atom (5‐28% of all metabolites), and to a lesser extent with two (0‐7%) or three (0‐2%) ^12^C atoms. At least half of the carbon atoms in each metabolite analyzed consisted of ^13^C. However, monoisotopic ^12^C compounds were absent from the internal standard. The underlying calculations can be found in Supplemental File 3 (“Evaluation of_12C Contamination”).

#### Quantification of metabolite concentrations in the internal standard using commercial ^12^C compounds

5 Prepare calibration mixtures with ^12^C commercial compounds in duplicate according to Table 6.
  Intracellular metabolite concentrations typically range from micromolar to millimolar. The chosen range of concentrations of commercial compounds for the quantification of metabolites in the internal standard are therefore designed to span this range. If the concentration of any metabolites in the internal standard deviates too much from this range, a new calibration should be performed for those metabolites, with the concentrations of commercial compounds adjusted to an appropriate higher or lower range.

**Table 6:** Concentration (µM) of the Commercial Compounds of Interest in the Six Calibration Mixes (S1‐S6)

	NaAD	NR	NMN	NAM	NAD	NADP	NADH	ADP	NA	NADPH	NaMN	ATP	Trp
S1	0.1	1	5	5	500	50	50	250	25	10	1	500	50
S2	0.5	5	10	50	1000	100	100	500	50	25	5	1000	75
S3	1	10	25	100	2000	200	200	1000	100	50	10	2000	100
S4	2	20	50	250	3000	300	300	2500	150	75	15	3000	150
S5	3.5	35	75	400	4000	400	400	4000	200	120	20	4000	200
S6	5	50	100	500	5000	500	500	5000	250	150	25	5000	250

6 Spike each calibration mixture with 100 µl ^13^C‐labeled yeast extract.
  The volume of spiked ^13^C‐labeled yeast extract was optimized to meet the detection limit of the mass spectrometer. Although the volume of internal standard used should be minimized to reduce cost, ^13^C‐labeled metabolites should be clearly detectable by the mass spectrometer.
7 Analyze the samples as described in Basic Protocol 3, steps 1‐16.
8 Create extracted ion chromatograms of the metabolites of interest using a ±10 ppm error marge.
9 Integrate the area of the ^12^C metabolites and of the ^13^C‐labeled metabolites.
  For NMN, a strong signal for its in‐source fragment was observed (m/z 211.0008 for the fully ^12^C‐labeled variant and m/z 216.0181 for the fully ^13^C‐labeled variant). Because this fragment yielded a stronger signal than the intact compound, it was used in creating the calibration curve.
10 Calculate the ^12^C/^13^C area ratio for each metabolite of interest and plot it against the known concentration of the ^12^C commercial standards (see example in Fig. 6).

**Figure 6:**
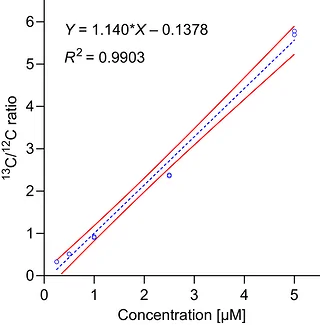
LC‐MS‐based quantification of ADP concentration in custom‐made internal standard. The internal standard is mixed at fixed volume ratio with solutions of unlabeled commercial standards covering a broad concentration range (Table 7). The ratio of ^12^C to ^13^C high‐resolution mass spectrometry signals measured in these calibration mixtures is plotted against the known ADP concentration in the unlabeled commercial standards. The red lines represent the 95% confidence interval. This figure was generated using GraphPad Prism version 11.0.2.

11 The linearity of this correlation for each metabolite can be calculated (Table 7).

**Table 7:** Linearity of the Calibration Curves Over a Specified Concentration Range

Compound	Concentration range (µM)	Linearity (*R* ^2^)
ATP	500‐5000	0.956
ADP	250‐5000	0.988
NADPH	10‐150	0.956
NADP	50‐500	0.968
NADH	50‐500	0.916
NAD	500‐5000	0.974
NaAD	0.1‐5	0.987
NR	5‐50	0.986
NAM	5‐500	0.981
NA	25‐250	0.989
Trp	50‐250	0.965
NMN	5‐100	0.988
NaMN	1‐25	0.970

12 Identify the concentration corresponding to a ^12^C/^13^C ratio of 1, which represents the metabolite concentration in the ^13^C yeast extract (see Table 8).
  If a ratio of 1 lies outside the calibration curve, adjust the concentration ranges of the commercial ^12^C compounds (Table 6) and generate new calibration curves.
  The linearity of most of the compounds identified in the internal standard is acceptable for metabolomics with a R^2^ of > 0.98 (Table 7). The linearity of NADH is rather low, which may be due to the difficulty of measuring the redox cofactors NAD(P)(H). As a result, we cannot confidently assign the NADH concentration to the internal standard (Table 8); however, the internal standard can still be used to show differences in NADH concentrations between conditions.

**Table 8:** Quantification of Metabolite Concentration in Internal Standards Using Calibration with Commercial Compounds

Compound	Concentration (µM)	95% confidence interval range	RT (min)*^a^*
ATP	2279.3	1986‐2571	10.0 ± 0.6
ADP	908.0	726‐1076	9.9 ± 0.4
NADPH	24.2	11‐35	12.5 ± 0.1
NADP	135.1	105‐161	13.7 ± 0.2
NADH	52.3	0‐108	15.3 ± 0.1
NAD	1151.5	865‐1396	16.5 ± 0.2
NaAD	0.4	0‐1	13.2 ± 0.2
NR	6.5	4‐9	14.8 ± 0.2
NAM	110.5	91‐129	9.9 ± 0.4
NA	123.5	117‐130	5.4 ± 0.7
Trp	78.4	63‐91	15.0 ± 0.2
NMN	11.6	8‐15	7.5 ± 0.1
NaMN	8.4	7‐10	6.0 ± 0.6

^*a*^ The reported retention time (RT) represents the average of the identified retention times for the fully 13C‐labeled metabolites across all calibration samples. The ± value indicates the observed RT range. A 95% confidence interval was used.

## Basic Protocol 7 GC‐MS CHARACTERIZATION OF THE INTERNAL STANDARD

Similar to Basic Protocol 6 using LC‐MS, this protocol describes how to quantify the glycolytic intermediates and amino acid concentrations in the internal standard by GC‐MS. Calibration curves with commercial ^12^C standards are used to quantify the concentration of each compound of interest in the internal standard. The lyophilization, derivatization, and GC‐MS analysis steps are the same as in Basic Protocol 4. The deviations from Basic Protocol 4 are in the sample preparation step and the final quantification of the internal standard. When Basic Protocol 7 is completed successfully, the internal standard will be quantified and ready to be used as internal standard for future metabolomics studies of NAD(P) metabolites (see Basic Protocol 8).

### Materials

See Basic Protocol 4

1 Prepare stock solutions for each commercial compound in ultrapure water as described in Basic Protocol 4, step 1.
2 From these stock solutions, prepare a calibration mix containing all targeted metabolites in known concentrations (Tables 9 and 10).
  The concentrations in the calibration mix are the same as those shown for CAL8 in Tables 9 and 10.

**Table 9:** Concentrations of the ^12^C Commercial Compounds in the Glycolytic Intermediate Calibration Mixture

CAL no.	1	2	3	4	5	6	7	8	9	10
Fum	0.15	0.30	0.75	1.49	2.98	5.96	8.94	14.91	29.81	59.63
Succ	0.25	0.49	1.23	2.46	4.93	9.85	14.78	24.63	49.26	98.53
Mal	0.25	0.49	1.24	2.47	4.95	9.89	14.84	24.73	49.46	98.92
aKG	0.15	0.30	0.74	1.48	2.97	5.94	8.91	14.84	29.69	59.38
G3P	0.12	0.25	0.62	1.25	2.50	5.00	7.49	12.49	24.98	49.97
GAP	0.01	0.02	0.06	0.12	0.24	0.49	0.73	1.22	2.45	4.90
Glc	0.49	0.99	2.47	4.93	9.87	19.74	29.61	49.34	98.69	197.37
Cit	0.49	0.98	2.46	4.91	9.83	19.65	29.48	49.13	98.25	196.51
iCit	0.04	0.07	0.19	0.37	0.75	1.49	2.24	3.74	7.47	14.95
2PG	0.04	0.08	0.19	0.38	0.75	1.50	2.25	3.76	7.51	15.03
DHAP	0.10	0.20	0.49	0.98	1.96	3.92	5.88	9.80	19.60	39.19
3PG	0.38	0.75	1.88	3.76	7.51	15.02	22.54	37.56	75.12	150.25
E4P	0.01	0.02	0.06	0.12	0.24	0.49	0.73	1.22	2.45	4.89
Rib5P	0.10	0.20	0.49	0.98	1.96	3.92	5.89	9.81	19.62	39.24
Ribu5P	0.05	0.10	0.21	0.52	1.05	2.11	3.17	5.29	10.58	21.17
Xyl5P	0.08	0.15	0.38	0.76	1.52	3.05	4.58	7.64	15.27	30.54
M6P	0.13	0.25	0.63	1.25	2.51	5.01	7.52	12.53	25.07	50.13
F6P	0.10	0.20	0.50	0.99	1.98	3.96	5.95	9.91	19.82	39.64
G6P	0.40	0.80	2.00	4.01	8.02	16.03	24.05	40.08	80.17	160.33
6PG	0.08	0.15	0.38	0.76	1.51	3.03	4.54	7.57	15.15	30.29
S7P	0.24	0.49	1.22	2.43	4.86	9.73	14.59	24.32	48.64	97.28
Tre	4.94	9.88	24.69	49.39	98.77	197.55	296.32	493.87	987.74	1975.48
FBP	0.50	1.00	2.51	5.01	10.02	20.05	30.07	50.11	100.23	200.46
T6P	0.29	0.58	1.44	2.88	5.76	11.51	17.27	28.78	57.56	115.11

Concentrations are given in µM. CAL 8 corresponds to the concentration for the aliquoted calibration mixture.

**Table 10:** Concentrations of the ^12^C Commercial Compounds in the Amino Acid Calibration Mixture

CAL no.	1	2	3	4	5	6	7	8	9	10
Ala	2.19	4.38	10.94	21.88	43.75	87.51	131.26	218.77	437.53	875.07
Gly	0.28	0.57	1.42	2.84	5.68	11.36	17.04	28.40	56.80	113.60
Val	0.51	1.01	2.53	5.06	10.12	20.25	30.37	50.62	101.24	202.48
Leu	0.06	0.12	0.30	0.60	1.20	2.40	3.60	6.00	12.00	24.00
Ile	0.06	0.12	0.31	0.61	1.22	2.44	3.66	6.11	12.21	24.43
Pro	0.18	0.37	0.91	1.83	3.65	7.30	10.95	18.25	36.50	73.01
Ser	0.51	1.02	2.54	5.09	10.17	20.35	30.52	50.86	101.73	203.45
Thr	0.51	1.01	2.54	5.07	10.15	20.29	30.44	50.73	101.46	202.91
Met	0.06	0.12	0.29	0.59	1.17	2.34	3.51	5.86	11.71	23.42
Asp	1.74	3.48	8.69	17.39	34.78	69.56	104.33	173.89	347.78	695.57
Phe	0.04	0.08	0.20	0.40	0.80	1.61	2.41	4.02	8.04	16.08
Cys	0.06	0.12	0.30	0.60	1.19	2.38	3.57	5.95	11.91	23.81
Glu	3.97	7.94	19.85	39.70	79.39	158.79	238.18	396.97	793.94	1587.88
Lys	0.24	0.48	1.19	2.38	4.76	9.52	14.28	23.81	47.62	95.23
Asn	0.25	0.49	1.23	2.46	4.91	9.83	14.74	24.57	49.14	98.28
Gln	2.03	4.06	10.14	20.28	40.56	81.12	121.68	202.79	405.59	811.18
Tyr	0.06	0.12	0.30	0.59	1.18	2.37	3.55	5.92	11.85	23.69
His	0.24	0.48	1.20	2.40	4.81	9.62	14.42	24.04	48.08	96.16
Trp	0.05	0.10	0.25	0.50	0.99	1.99	2.98	4.97	9.94	19.89

Concentrations are given in µM. CAL 8 corresponds to the concentration for the aliquoted calibration mixture.

3 Prepare aliquots from this calibration mix and store at −80°C.
4 Prepare calibration samples 1‐10 (CAL1‐CAL10) following the pipetting scheme (Table 11). The range of concentrations of the ^12^C compounds is displayed in Tables 9 and 10.
  Calibration samples are prepared by adjusting the added volume of calibration mix according to the target concentration (Table 11). Lower volumes are used for low‐concentration calibration samples (CAL 1‐7) and higher volumes for high‐concentration calibration samples (CAL 9‐10). The use of larger volumes is compatible with the subsequent lyophilization step, which removes excess solvent and standardizes final sample concentrations.
  For calibration samples with an initial volume <100 µl (corresponding to low concentrations of commercial compounds; Table 11), 200 µl of water was added before freezing to prevent defrosting during the freeze‐drying.

**Table 11:** Pipetting Scheme to Obtain Calibration Mixes with a Broad Range of Metabolite Concentrations

Calibration name	Calibration mix (µl)	NaCl*^a^* (µl)	Water*^b^* (µl)	Internal standard (µl)
CAL1	1	30	200	10
CAL2	2	30	200	10
CAL3	5	30	200	10
CAL4	10	30	200	10
CAL5	20	30	200	10
CAL6	40	30	200	10
CAL7	60	30	200	10
CAL8	100	30	−	10
CAL9	200	30	−	10
CAL10	400	30	−	10

^*a*^ NaCl (100 mg/ml) is added only for the amino acid standard and must be omitted from the glycolytic intermediate standards. *^b^* Ultrapure water.

5 Lyophilize samples CAL1‐CAL10 (see Basic Protocol 4).
6 Derivatize samples CAL1‐CAL10 (see Basic Protocol 4).
7 Analyze samples CAL1‐CAL10 (see Basic Protocol 4).
8 Mass Hunter analysis software (version 12.0/12.1; Agilent) can be used for data processing.
9 Integrate signals and calculate concentrations based on the calibration line established according to Table 11.
10 Calibration graphs are constructed based on peak area ratios of the natural ^12^C peak areas of targeted metabolites to the peak areas of the respective ^13^C‐labeled internal standards versus the concentration of the commercial natural ^12^C compound in the calibration mixes. Use 1/*y* ^2^ weighting for the construction of calibration curves. The calibration curve for glucose‐6‐phosphate is shown in Figure 7 as an example.

**Figure 7:**
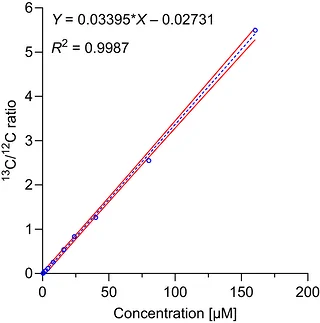
GC‐MS‐based quantification of glucose‐6‐phosphate concentration in the homemade internal standard. The internal standard is mixed at fixed volume ratio with solutions of unlabeled commercial standards covering a broad concentration range (Table 9). The ratio of ^12^C to ^13^C high‐resolution mass spectrometry signals measured in these calibration mixtures is plotted against the known glucose‐6‐phosphate concentration in the unlabeled commercial standards. Measurements were performed in analytical duplicates. The calibration curve was generated using nonlinear regression with 1/*y* ^2^ weighting. The red lines represent the 95% confidence interval. This figure was generated using GraphPad Prism version 11.0.2.

11 The concentration of the compound in the ^13^C yeast extract is calculated from the calibration curves using equation 5.

(5)

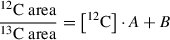

where A and B are the slope and intercept of the calibration curve, respectively; ^12^C area and ^13^C area are the peak areas of unlabeled (^12^C) and labeled (^13^C) metabolites in the calibration mix, respectively; and [^12^C] is concentration of the ^12^C metabolite

Slope A of the calibration curve is obtained by linear regression, giving equation 6.

(6)

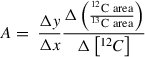




When working with an internal standard, the response factors of both the compound of interest and the internal standard are needed for the correction. However, as the ^12^C and ^13^C variants of a compound have the same chemical composition, their response factor (Rf) is the same. In this case Rf = 1.

The intercept B of the calibration curve reflects contributions from residual ^12^C metabolites or other matrix components present in the internal standard. Because we match the internal standard concentrations in both the calibration mixes and the samples, the relative contribution of ^12^C contamination and matrix effects is expected to be equivalent. Consequently, to simplify the calculations, the intercept was assumed to be consistent and omitted.

The concentration of the ^13^C compounds in the internal standard were obtained using equation 5, 6, and 7.

(7)

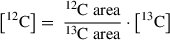




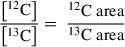




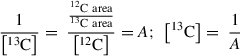




See Table 12 for concentrations of glycolytic intermediates and Table 13 for concentrations of amino acids in the internal standard. The concentrations are given for 100 µl of internal standard.

**Table 12:** Quantification of Glycolytic Metabolite Concentrations in the Internal Standard Using Calibration with Commercial Compounds

Compound	Concentration (µM)	Slope	Intercept	Linearity (*R* ^2^)
Fum*^a^*	0.94	1.066	0.212	0.90
Succ*^a^*	0.82	1.219	0.494	0.90
Mal*^a^*	8.96	0.112	0.012	0.94
aKG	1.86	0.538	0.021	0.92
GAP	0.22	4.568	3.573	0.76
Glc*^a^*	3.84	0.261	0.485	0.96
Cit*^a^*	260.96	0.004	0.008	0.89
iCit	6.65	0.150	0.001	0.99
2PG	1.94	0.515	−0.005	0.93
DHAP	1.06	0.940	0.082	0.91
3PG	20.05	0.050	0.005	0.95
E4P	0.04	25.442	8.540	0.68
Rib5P	3.04	0.329	0.001	0.92
Ribu5P	1.04	0.965	0.099	0.76
Xyl5P	1.31	0.763	0.012	0.97
M6P	11.70	0.085	0.002	0.91
F6P	7.24	0.138	−0.001	0.93
G6P	34.00	0.029	0.002	0.92
6PG	3.03	0.330	−0.015	0.85
S7P	10.58	0.094	−0.005	0.92
Trehalose	626.57	0.002	0.003	0.96

^*a*^ The concentrations of these compounds were calculated from injections measured in split (1:5) mode.

The reported concentrations have been corrected for the addition of 10 µl of internal standard to the calibration sample. All samples were measured in duplicate.

**Table 13:** Quantification of Amino Acid Concentrations in the Internal Standard Using Calibration with Commercial Compounds

Compound	Concentration (µM)	Slope	Intercept	Linearity (*R* ^2^)
Alanine	112.98	0.089	−0.035	0.985
Glycine	27.19	0.368	0.195	0.981
Valine	116.73	0.086	−0.006	0.988
Leucine	17.76	0.563	0.004	0.990
Isoleucine	33.49	0.299	0.011	0.989
Proline	6.11	1.637	−0.041	0.982
Serine*^a^*	76.28	0.131	0.001	0.999
Threonine	61.46	0.163	−0.012	0.987
Methionine*^a^*	1.27	7.886	0.143	0.981
Aspartate	266.64	0.038	−0.012	0.985
Phenylalanine*^a^*	24.81	0.403	0.001	0.987
Glutamate	1967.34	0.005	0.007	0.984
Lysine	415.89	0.024	0.003	0.983
Asparagine	125.63	0.080	−0.005	0.982
Glutamine	901.14	0.011	0.004	0.986
Tyrosine*^a^*	61.21	0.163	0.022	0.991
Histidine	374.45	0.027	−0.002	0.979
Tryptophan*^a^*	22.27	0.449	0.024	0.991

^*a*^ The concentrations of these compounds were calculated from splitless injections.

The reported concentrations have been corrected for the addition of 10 µl of internal standard to the calibration sample. All samples were measured in duplicate.

For the glycolytic intermediates, the metabolites GAP, E4P, Ribu5P, and 6PG could be detected by GC‐MS analysis; however, the concentrations were too low for accurate quantification. These sugar phosphates and high‐molecular‐mass molecules are often more difficult to measure because they are more sensitive to interruptions in the lyophilization process, exposure to sunlight, and matrix effects (Niedenführ et al., 2016).

The reported concentration has been corrected for the addition of 10 µl of internal standard to the calibration sample. All samples were measured in duplicate.

## Basic Protocol 8 QUANTIFICATION OF METABOLITES IN YEAST SAMPLES

Once the internal standard has been characterized as described in Basic Protocols 6 and 7, it is ready to be used for quantification of intracellular metabolites in cell extracts. This protocol describes how to perform cultures for quantification of intracellular metabolites using internal standards.

### Materials

*S. cerevisiae* strain IMX2600 (van den Broek et al., 2024) or other strain of interest in 30% (v/v) glycerol (1 ml)
Methanol (HPLC grade, >99.9%), prechilled to −40°C
Synthetic mineral medium supplemented with 2% glucose and vitamin solution (Verduyn et al., 1992) or other growth medium of interest
83% (v/v) methanol in demineralized (demi) water, −40°C
75% (v/v) ethanol
Internal standard (Basic Protocols 6 and 7)

500‐ml round‐bottom shaker flasks
Innova 4000 incubator shaker (Eppendorf) at 30°C
Jenway 7200 scanning spectrophotometer (Cole‐Parmer Inc., Chicago, USA)
15‐ml Greiner tubes
Scale
Freezer, −40°C
Pipetboy
2‐ml pipets, sterile
Vortex
Cryostat with cryofluid (70% [v/v] ethylene glycol), set at −40°C
Tabletop centrifuge with rotor for 15‐ml tubes, −19°C
Waste bucket for methanol waste (plastic and liquid)
5‐ and 20‐ml‐capacity bottle‐top dispensers
Water baths, 75°C and 95°C
Standard 14‐mm‐diameter marbles
Benchtop centrifugal vacuum concentrator (SpeedVac): e.g., Labconco, Eppendorf, or equivalent, with vacuum pump and cold trap, capable of evaporating ethanol and equipped with a rotor for 15‐ml conical tubes
Syringes
0.2‐µm‐pore‐size PVDF syringe filters
Nitrile gloves

*CAUTION*: Methanol is a neurotoxic compound and can penetrate the skin. Always wear (nitrile) gloves when handling methanol and work as much as possible in the fume hood. Try to minimize the steps performed outside the fume hood.

1 Grow the strain of interest in a 500 ml round‐bottom shake flask or bioreactor in synthetic mineral medium with 2% glucose and vitamin solution.
  Bioreactors are preferred for intracellular metabolite analysis as parameters (pH, gases, nutrients) can be tightly controlled, including (limited) nutrient supply. Additionally, bioreactors such as those used in the present study can be equipped with sampling ports enabling ultrafast sampling within milliseconds (Fig. 2), which minimizes metabolic changes during sampling. However, as described in this protocol, sampling from shake flask cultures is also possible, provided that gas and nutrients are not in limited supply for cells during sampling.
2 Weigh 5 ml methanol into a 15‐ml Greiner tube and record the exact weight. Store at −40°C.
3 Record the optical density of the culture.
4 Once the culture reaches the desired optical density, sample 1.2 ml of culture into 5 ml of −40°C methanol as quickly as possible using a Pipetboy with a 2‐ml pipet.
  To be above the detection limit of the mass spectrometer, the sampling volume must be adjusted to the cell density of the culture and the expected intracellular metabolite levels. The sampling volume may be decreased or increased as long as the quenching ratio of 1:5 (culture: methanol) is maintained.
5 Immediately vortex the sample.
6 Weigh the broth/methanol sample and record the exact weight. Keep the sample cold on dry ice or in a −40°C cryostat.
  As mentioned previously, to arrest all metabolic activity, the samples should be kept cold until metabolite extraction using boiling ethanol.
7 Perform sample processing and metabolite extraction according to Basic Protocol 2, steps 7‐34, with the following adjustments:
  a. In step 9, add 5 ml instead of 20 ml of 83% (v/v) methanol (−40°C).
  b. Skip step 12 because Basic Protocol 8 only uses 15‐ml Greiner tubes and therefore the rotor does not need to be changed.
  c. Skip step 13 because the second washing step will use 5 ml of 83% methanol again.
  d. In step 15, add 5 ml instead of 3 ml of 83% (v/v) methanol (−40°C).
  e. Skip steps 17‐19 because the transfer from 50‐ to 15‐ml tubes is not needed.
  f. After step 21, add internal standard to the cell suspension which is mandatory for quantification of intracellular metabolites. Additionally, [^13^C]NA was spiked at a final concentration of 100 µM since this compound is not present in the internal standard.
  g. In step 25, boiling for 3 min is sufficient.
  h. In step 29, we have used a Milli‐Q water volume of 600 µl to resuspend the pellets and found that this resulted in a measurable metabolite concentration for *S. cerevisiae* cultures grown on chemically defined medium with glucose as sole carbon source during exponential growth (OD_660_ 2‐8)
    In this protocol, 100 µl of internal standards prepared in Basic Protocol 5 are spiked into each tube. The volume of internal standard to add is optimized to meet the detection limit of the mass spectrometer. While the volume of internal standard used should be minimized to reduce cost, ^13^C‐labeled metabolites should be clearly detectable by the mass spectrometer.
8 Perform LC‐ or GC‐MS analysis following Basic Protocol 6 or 7.
9 See the Understanding Results section for details of how to calculate the intracellular metabolite concentrations based on mass spectrometry output.

## REAGENTS AND SOLUTIONS

### Ammonium bicarbonate, 10 mM in 60% acetonitrile (50 ml)

Weigh 39.53 mg ammonium bicarbonate and add to 30 ml Optima LC‐MS‐grade acetonitrile and 20 ml Optima LC‐MS‐grade water in a 50‐ml Falcon tube. Store up to 6 months at 4°C.

### Antifoam C solution, 1/10

Add 10 ml antifoam C emulsion (shake well before use) to a bottle. Add 90 ml demineralized (demi) water and shake well. Sterilize by autoclaving at 121°C for 20 min. Store up to 1 year at 4°C.

### Batch salt base (350 ml)

Calibrate the pH probe using pH 4 and pH 7 buffers. Add a stir bar to a 500‐ml Schott bottle (500 ml). Fill the bottle with 350 ml demi water and mark the volume. Remove ∼100 ml demi water from the bottle. Put the bottle on a magnetic stirrer plate.

Dissolve the following compounds in the water:

• 0.5 g ammonium sulfate [(NH_4_)_2_SO_4_]
• 1.5 g of ammonium dihydrogen phosphate (NH_4_H_2_PO_4_)
• 1.76 g of potassium dihydrogen phosphate (KH_2_PO_4_)
• 0.5 g magnesium sulfate heptahydrate (MgSO_4_•7H_2_O)
• 1 ml trace element solution
• 0.75 ml antifoam C (1/10) solution
Adjust the pH to 5 with 2 M KOH.
Adjust the volume to 350 ml.
Sterilize by autoclaving at 121°C.
Store up to 1 year at room temperature.
  CAUTION: When working with high‐molarity acids and bases, please take the required precautions, including wearing protective clothing, glasses, and gloves.

### Fed‐batch medium (640 ml)

NOTE: It is recommended that the fed‐batch medium be prepared and filter sterilized on the day it will be used.

Calibrate the pH probe using pH 4 and pH 7 buffers. Add 1 ml of 1/10 antifoam C solution (see recipe) to a 1‐liter Schott bottle. Sterilize the bottle by autoclaving at 121°C.

Fill a nonsterile Schott bottle (1 liter) with 640 ml demi water and mark the volume. Remove ∼100 ml demi water from the bottle. Place the bottle on a magnetic stirrer plate and add a stir bar.

Dissolve the following compounds in the medium:

• Either 74.52 g [^12^C]glucose monohydrate (trial run) or 70 g [^13^C]glucose anhydrous (real run)
• 0.64 g ammonium sulfate [(NH_4_)_2_SO_4_]
• 1.92 g ammonium dihydrogen phosphate (NH_4_H_2_PO_4_)
• 2.24 g potassium dihydrogen phosphate (KH_2_PO_4_)
• 0.64 g magnesium sulfate heptahydrate (MgSO_4_•7H_2_O)
• 3.2 ml trace element solution (see recipe)
• 3.2 ml vitamin solution (see recipe)

Adjust the pH to 5 with KOH (2 M). Remove the stir bar. Adjust the volume to 640 ml. Filter sterilize using a bottle‐top filter and the vacuum pump into the sterilized bottle containing anti‐foam C solution.

CAUTION: When working with high‐molarity acids and bases, please take the required precautions, including wearing protective clothing, glasses, and gloves.

### Glucose solution (100 ml)

Place a stir bar in a 250‐ml Schott bottle, fill the Schott bottle with 100 ml Milli‐Q‐purified water, and mark the volume. Remove ∼20 ml Milli‐Q water. Place the bottle on a magnetic stirrer plate. Dissolve 6.81 g of [^12^C]glucose monohydrate (trial run) or 6.39 g [^13^C]glucose anhydrous (real run) in the water.

Anhydrous [^13^C]glucose may dissolve poorly. If needed, to speed up its dissolution, put the solution in a 37°C water bath.

Once the glucose has dissolved, adjust the volume to 100 ml. Sterilize the glucose solution at 110°C. Store up to 1 year at room temperature.

### Mobile phase A/weak wash: 50 mM ammonium bicarbonate in MS‐grade H_2_O (1 liter)

Weigh out 3.953 g of ammonium bicarbonate and add to 1 liter of Optima LC‐MS‐grade H_2_O. Store up to 4 months at room temperature.

### pH titration solution (base; 4 M NH_4_OH; 200 ml)

CAUTION: NH_4_OH has high vapor pressure and the gas is toxic. Always work in the fume hood and do not sterilize this solution. Wear protective clothing, glasses, and gloves.

Add 88 ml Milli‐Q‐purified water to a 250‐ml Schott bottle. Sterilize this bottle by autoclaving at 121°C. Sterilize the screw cap with the two inlets/outlets separately in a bottle. Working in a fume hood, add 112 ml NH_4_OH. Transfer the sterilized screw cap with three distributors to the base solution bottle. Store up to 1 year at 4°C.

### Strong wash/seal wash: 10% acetonitrile/10 mM ammonium bicarbonate in H_2_O (1 liter)

Weigh 0.791 g of ammonium bicarbonate and place in a 1‐liter flask.
Add 900 ml Optima LC‐MS‐grade H_2_O to the flask.
Add 100 ml Optima LC‐MS‐grade acetonitrile to flask.
Store up to 1 year at room temperature.

### Trace element solution (5 liters)

Calibrate the pH probe using pH 4 and pH 7 buffers. Place a stir bar in a 5‐liter flask. Fill the flask with demi water and mark the 5‐liter level. Remove ∼1.25 liter of water. Put the flask on a magnetic stirrer plate. Dissolve 75 g EDTA and 22.5 g zinc sulfate heptahydrate (ZnSO_4_•7H_2_O) in the water. Adjust the pH to 6.0 with 1 M NaOH.

Add all the following compounds one by one in the specified order, correcting the pH to 6.5 after each addition. Be sure to wait until each compound has completely dissolved before adding the next one.

• 4.2 g manganese (II) chloride•2H_2_O
• 1.5 g cobalt (II) chloride•6H_2_O (*CAUTION*: toxic)
• 1.5 g copper (II) sulfate•5H_2_O
• 2 g disodium molybdate•2H_2_O
• 22.5 g calcium chloride•2H_2_O
• 15 g iron sulfate•7H_2_O
• 5 g boric acid
• 0.5 g potassium iodide

When all compounds have dissolved, adjust the pH to 4.0 with 1 M HCl. Add demi water to adjust the volume to 5 liters. Store up to 6‐12 months at 4°C.

CAUTION: When working with high‐molarity acids and bases, please take the required precautions, including wearing protective clothing, glasses, and gloves.

### Vitamin solution (1 liter)

Calibrate the pH probe using pH 4 and pH 7 buffers. Add a stir bar to a 1‐liter Schott bottle. Fill the Schott bottle with 1000 ml demi water and mark the volume. Remove ∼200 ml demi water from the bottle.

Dissolve 0.05 g of d‐biotin in 10 ml of 0.1 M NaOH in a Greiner tube. Add the biotin solution to the 800 ml water in the Schott bottle.

Place the bottle on a magnetic stirrer plate. Adjust the pH to 6.5 with 1 M HCl. Add 1 g thiamine and dissolve properly. Adjust the pH to 6.5 with 1 M NaOH. Adjust final volume to 1 liter. Filter sterilize the vitamin solution using a 0.22‐µm‐pore‐size syringe filter and store up to 3 months at 4°C.

## COMMENTARY

### Critical Parameters and Troubleshooting

Table 14 lists problems that may arise with this procedure along with their possible causes and solutions.

**Table 14:** Troubleshooting

Problem	Possible cause	Solution
Production of ethanol or other fermentation products during the fed‐batch phase	Feed rate too close to or above µ_max_	Decrease feed rate
	Insufficient oxygen supply	Increase stirring speed or sparging
Foaming	Intensive gas sparging and stirring can lead to foam formation in the presence of some medium components or molecules produced by the microorganism	Increase anti‐foam addition Decrease stirring/sparging if oxygen supply is sufficient
A fraction of the metabolites in the internal standard is fully unlabeled	The carbon of some (unlabeled) nutrients such as amino acids or vitamins can be incorporated into biomass, leading to a ^12^C fraction in the biomass	• If not essential, remove the unlabeled compound from the medium • If essential, add a ^13^C‐labeled variant of the required medium component
	If the batch phase is started with a large amount of (unlabeled) biomass, unlabeled metabolites will contaminate the internal standard	Start the batch with a small amount of biomass (a single 1‐ml aliquot of cells stored in glycerol is advised in this protocol)
	If there are too few generations between the inoculation and the final sampling for internal standard, a substantial fraction of metabolites will be fully unlabeled	Make sure that the nutrients in the medium and oxygen supply support ≥12 generations
Loss of metabolites (leakage) during the sample processing	Quenching not suitable for the organism of interest	Optimize quenching method
	Temperature is not kept low enough during the sample processing	Keep the sample cold enough using a cryostat or −40°C freezer
Low concentration of metabolites in the cell extract	Metabolite loss during sample processing (see previous point)	
	The addition of water to dissolve the metabolite extract was too high	Concentrate the dissolved metabolite extract
Concentrations in the calibration curves are too far removed from the concentration in the internal standard	The concentrations of certain metabolites were under‐ or overestimated	Repeat the calibration with an adjusted concentration range that matches the intracellular metabolite concentration.
No CO_2_ measurements during the “real” run	[^13^C]CO_2_ measurements are less sensitive Gas analyzer cannot detect [^13^C]CO_2_	Use a mass spectrometry‐based gas analyzer
Metabolites of interest are not present in the internal standard	The internal standard was produced under conditions different from those for the sample being analyzed	• Use growth conditions that lead to the synthesis of the metabolites of interest • Spike samples with a known concentration of a commercially available ^13^C‐labeled molecule
	An organism different from the one used to produce the internal standard is used	Spike samples with a known concentration of a commercially available ^13^C‐labeled molecule

### Understanding Results

This section explains how to obtain intracellular metabolite concentrations by processing the data generated in Basic Protocol 8. The calculations can be performed using Supplemental File 4 (an Excel file). For more detailed information on these calculations, please refer to Wahl et al. (2014).

1. Visualize and manually perform quality control of the mass spectrometry data using XCalibur or other similar software.

2. For each metabolite, extract the ^12^C and ^13^C peak intensities. We used a Matlab script to extract the peak intensities, and the Matlab script can be provided upon request.

3. Calculate the ^12^C/^13^C ratio using equation below or use the spreadsheet “Ratio” in Supplemental File 4.


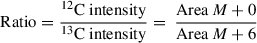




4. Enter the slope, intercept and quality of fit of the calibration curves in the spreadsheet “Calibration Curves” in Supplemental File 4.

5. Use the ^12^C/^13^C ratio to calculate the measured amount of corresponding metabolite using the calibration equation (using the spreadsheet “Metabolite Amount LCMS Sample” in Supplemental File 4). This step includes a correction for final amount of water in which the metabolite extract was dissolved before the LC or GC‐MS analysis (20 µl).

6. Enter the sampled culture volume and corresponding optical density at 660 nm (OD_660_) in the spreadsheet “Growth Curves” in Supplemental File 4. The spreadsheet will convert the optical density into dry biomass (gDW) concentration at sampling time using a pre‐established OD_660_ dry‐weight correlation of yeast.

7. The spreadsheet “Metabolite Amount umol per gDW” in Supplemental File 4 multiplies the sampling volume by the dry‐weight concentration (g L^−1^) to obtain the weight of dried biomass used at the time of sampling for each sample. It will then correct the LC/GC‐MS‐quantified metabolite amount with this dry weight to generate the metabolite concentration expressed in µmol metabolite per gram biomass dry weight.

8. Finally, bring the metabolite concentration to µM by correcting with the intracellular volume per gram of dry biomass as described in the spreadsheet “Metabolite Amount uM” in Supplemental File 4. In yeast an intracellular volume of 1.7 ml per gram of dry weight is typically used. Figure 8 shows the intracellular concentrations of a subset of metabolites in nucleotides metabolism calculated in Supplemental File 4 using custom‐made internal standards. Samples were taken as described in Basic Protocol 8 from cultures in shake flasks.

**Figure 8:**
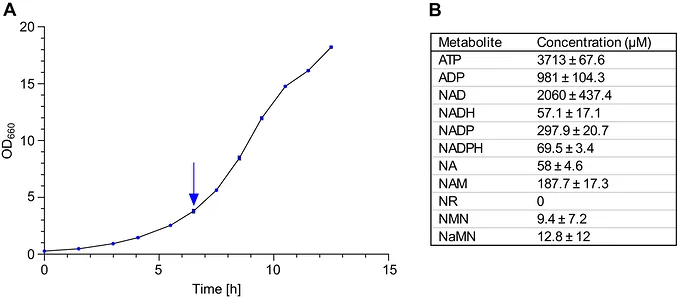
Intracellular metabolite concentrations in *Saccharomyces cerevisiae* cultures measured using custom‐made internal standards. *S. cerevisiae* IMX2600 (van den Broek et al., 2024) was grown in aerobic shake‐flask chemically defined medium with glucose as sole carbon source. (**A**) Biomass profile. (**B**) Intracellular metabolites concentrations at time point 6.5 hr. Data represent the average and standard deviation of three independent culture replicates.

Nicotinamide riboside is an unstable compound that rapidly degrades during sample processing. It can be detected, but the measured concentrations are unreliable.

The measured NADPH/NADP⁺ ratio was outside of the expected range (expected ∼1; see, e.g., Zhang et al., 2015), although both compounds providing a strong, quantifiable signal. Despite careful operation of the column (properly grounded, thoroughly cleaned and stored) and the prevention of redox‐active additives (e.g., formic acid), the conductive surface of the porous graphitic carbon (PGC) stationary phase might have affected the detection of certain redox‐sensitive analytes such as the NADP(H) couple (Bapiro et al., 2016; Melmer et al., 2010; Pabst et al., 2010; Törnkvist et al., 2004; van Ede et al., 2026).

### Time Considerations

As shown in Figure 1, the in‐house production and characterization of internal standards require the following time allocation for *S. cerevisiae*:

• ∼5 days for the batch phase (1 day of medium preparation, 1 day for autoclaving, 3 days (∼66 hr) of fermentation).
• ∼2 days for the fed‐batch phase of the fermentation (∼55 hr).
• 1 day for the sampling.
• 4 days for the extraction of metabolites, however the duration depends on the number of samples to process.

The quenching of the samples and further processing until after the boiling ethanol step for metabolite extraction (step 25 of Basic Protocol 2) is time sensitive as metabolites might be unstable if the samples are not strictly kept at low temperature close to 0°C.

The time required for ethanol evaporation depends on the capacity of the vacuum evaporator. Considering the standard capacity of a vacuum evaporator and the number of samples to process, this step will take multiple days.

• ∼4 days for quantification (1 day for preparation of calibration curve samples, 1 day for running all the samples, and 2 days for data analysis)

Note that the calibration curves and the amount of internal standard added to each sample might have to be optimized. It is essential to have a calibration curve in the correct range—around a ^12^C/^13^C ratio of 1—to prevent under‐ or overestimation of metabolite concentrations. As a results, consider that this step might take two or three iterations when the methods is used for the first time.

The above‐indicated time investment is for one successful run to produce, extract, and characterize the internal standard. Please keep in mind that you will have to go through this cycle at least two times to perform a trial run to test the yield of biomass on substrate and to identify whether the metabolites of interest are present in the cell extract. The trial run might have to be executed multiple times.

### Author Contributions

**Miriam Cammaert**: Conceptualization; data curation; formal analysis; investigation; methodology; validation; visualization; writing—review and editing. **Rosalie Wouters**: Conceptualization; investigation; visualization; writing—review and editing. **Jitske van Ede**: Data curation; investigation; formal analysis; methodology; software; validation; visualization; writing—review and editing. **Erik de Hulster**: Formal analysis; investigation; methodology; validation. **Christiaan Mooiman**: Formal analysis; investigation; methodology; validation. **Patricia van Dam**: Formal analysis; investigation; methodology; validation; writing—review and editing. **Martin Pabst**: Conceptualization; data curation; formal analysis; funding acquisition; investigation; methodology; project administration; software; supervision; validation; writing—review and editing. **Walter van Gulik**: Conceptualization; methodology; resources; validation; writing—review and editing. **Pascale Daran‐Lapujade**: Conceptualization; formal analysis; funding acquisition; methodology; project administration; supervision; validation; visualization; writing—original draft; writing—review and editing.

### Conflict of Interest

The authors declare no conflict of interest.

## Supporting information




*Supplemental File 1: Excel file; can be used to simulate the fed‐batch feeding profile to estimate the final biomass concentration and to determine the sampling points*.

*Supplemental File 2: Text file; describes how to calculate the fed‐batch feeding profile*.

*Supplemental File 3: Excel file; shows how to calculate the amount of ^12^C contamination in the internal standard*.

*Supplemental File 4; Excel file; can be used to calculate metabolite concentrations from mass spectrometry data*.

## Data Availability

All acquired data discussed here are publicly accessible through the 4TU.ResearchData platform, DOI 10.4121/89ba9ee3‐f072‐4dda‐97ba‐683cce975a3b. The LC‐MS data analysis MATLAB script is available upon request.
